# PathSys: integrating molecular interaction graphs for systems biology

**DOI:** 10.1186/1471-2105-7-55

**Published:** 2006-02-07

**Authors:** Michael Baitaluk, Xufei Qian, Shubhada Godbole, Alpan Raval, Animesh Ray, Amarnath Gupta

**Affiliations:** 1 San Diego Supercomputer Center, University of California San Diego, 9500 Gilman Drive, La Jolla, CA, 92093, USA; 2 Keck Graduate Institute, 535 Watson Drive, Claremont, CA, 91711, USA; 3 School of Mathematical Sciences, Claremont Graduate University, 710 N. College Ave, Claremont, CA 91711, USA

## Abstract

**Background:**

The goal of information integration in systems biology is to combine information from a number of databases and data sets, which are obtained from both high and low throughput experiments, under one data management scheme such that the cumulative information provides greater biological insight than is possible with individual information sources considered separately.

**Results:**

Here we present PathSys, a graph-based system for creating a combined database of networks of interaction for generating integrated view of biological mechanisms. We used PathSys to integrate over 14 curated and publicly contributed data sources for the budding yeast (*S. cerevisiae*) and Gene Ontology. A number of exploratory questions were formulated as a combination of relational and graph-based queries to the integrated database. Thus, PathSys is a general-purpose, scalable, graph-data warehouse of biological information, complete with a graph manipulation and a query language, a storage mechanism and a generic data-importing mechanism through schema-mapping.

**Conclusion:**

Results from several test studies demonstrate the effectiveness of the approach in retrieving biologically interesting relations between genes and proteins, the networks connecting them, and of the utility of PathSys as a scalable graph-based warehouse for interaction-network integration and a hypothesis generator system. The PathSys's client software, named BiologicalNetworks, developed for navigation and analyses of molecular networks, is available as a Java Web Start application at .

## Background

Complex networks of molecular and genetic interactions are increasingly being studied for insights into biological mechanisms [1-3]. Such studies include deciphering genome-wide protein-protein interactions [4]], large-scale analysis and prediction of gene regulatory networks [5], construction of metabolic pathways [6], and development of synthetic genetic interaction networks [7,8]. Here we collectively call these different networks *Molecular Interaction Graphs *(MIGs). The availability of MIGs has paved the way for the emergence of a new paradigm of biology in which networks of interactions are being analyzed for understanding of biological phenomena [3,9-12]. Truly integrated analyses across multiple databases of different functionalities are still rare yet promising [13]. Such advances underscore the need to develop information management frameworks for adequate modeling of graph-structured data and graph-oriented operations [14,15]. In the absence of an efficient information management system that allows biologists to query discrete and large databases simultaneously, the full potential for functional genomics resources will remain under-utilized.

Here we present PathSys as an information integration system, which integrates MIGs and ontologies and show how its integration engine can be used to address biologically relevant questions. We describe the capabilities of the system based upon our current Yeast Data Warehouse, where over 14 (for full list see Additional file or supplemental materials at [46]) curated and publicly contributed data sources for the budding yeast (*S. cerevisiae*) are integrated. The system architecture, however, is designed as a general-purpose tool for application to potentially any biological model.

### Related work

The most noted pathways source KEGG [16] has an API and XML schema that is centered on enzymatic activities in cellular process. No general ontology for representation of cellular events or description of biological entities exists. The KEGG ontology is organized around the concept of binary relation [17], defining relationships between database objects (such as the relationship between reactions, substrates and products; that between an enzyme and its location in the metabolic pathway; or that between an enzyme and a protein super family to which it belongs).

Karp *et al*. [18,19], in BioCyc, on the other hand define different types of molecules each with its own class, and consider different states of a molecule as different members within a class. Reactions are defined to be independent entities, and distinct relations, called slots, link molecules to the reactions. Each molecule may optionally be tagged with a cellular compartment. Their ontology makes use of the "pathway" concept to define summary abstractions, used for defining data at varying levels of detail. However, like KEGG, the BioCyc system implements a specific data model for its own application.

The PathDB [14] is a relational database developed for metabolic networks. Here the central element is a *biochem*, (e.g. RNA, DNA, Compound) which is used to build other *biochem *objects. The transition is modelled by the explicit representation of a biochemical reaction whose substrates, products, mediators with its kinetic properties are recorded. The Pathways database system [15] models pathways as a directed hypergraph where nodes represent pathway elements (substrates and products of a reaction). Pathways support queries where operations such as shortest path, unions and intersections of paths, and node-neighbourhoods can be performed. However, since they do not present a query language, the exact query capability of the system is unclear. Other works include those of Ochs *et al*. [20], who developed a metabolic map from a relational model of biochemical interactions, and of Bhalla [21], where a database of chemical reactions is mapped to a system of pathway graphs.

By contrast to KEGG or BioCyc, PathSys is based upon a generic graph model that can integrate any combination of graph data sources. Consequently it represents a wider range of data types and relationships and can be extended by including any new data source or ontology. Unlike any previous system, PathSys is a general-purpose graph warehouse with its own data definition and query language, augmented with biological data types, and hence can implement any specific graph-structured biological model. The benefit of having an integration platform such as PathSys is that it can be constructed over those databases that typically focus on specific interaction studies [22-24], as well as those of process-specific databases such as BioCyc and KEGG focusing on specific biological processes.

## Results

### The PathSys system

#### Architecture

**Figure 1:**
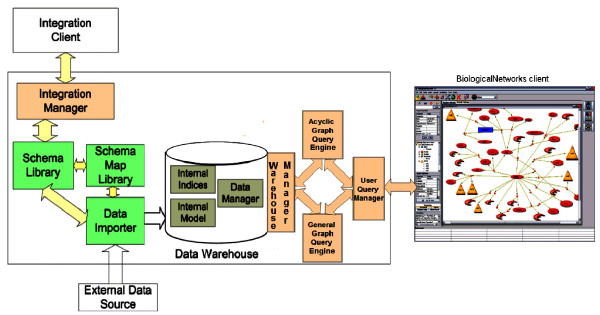
PathSys System architecture.

The system architecture of PathSys is shown in Figure 1. The system is designed around a warehouse that holds the data according to an internal schema (discussed in the next subsection), a number of specialized index structures that facilitate graph operations, and a *Data Manager *that keeps the data and external indices synchronized.

**Figure 2:**
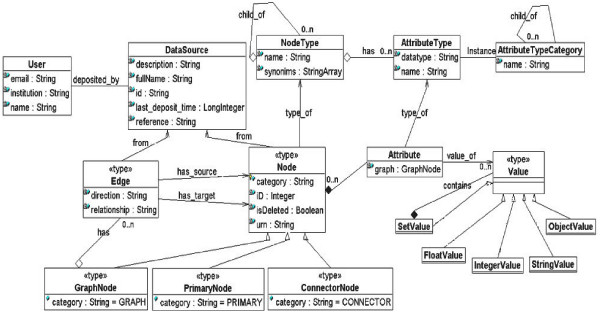
The UML diagram of the internal data model.

We consider two kinds of users. The first is a typical information systems person who creates a new integrated schema through the *Integration client*, to add a new data source to an existing integrated schema or to define new queries to support a specific kind of analysis. The process of adding a new data source is as follows. The user first determines that the data schema is specified in a language accepted by PathSys (e.g., a relational schema, an XML schema). Next, the schema is sent to PathSys, which validates it and stores in the *Schema Library*. The user then specifies the mapping between the schema element and the internal data model of PathSys described in Figure 2. Finally, it is stored in the *Schema Map Library *and the data are ingested into PathSys warehouse through the *Data Importer *much like the bulk loading operation in a standard DBMS.

The second category of users is a biologist who enters PathSys through the visualization client, BiologicalNetworks [47]. In one sense the visualization and graph manipulation capabilities of BiologicalNetworks are comparable to that of existing visual information integration systems such as Cytoscape [25] and VisANT [26,27] as well as commercially available tools such as GeneGO [28] and PathwayAssist [29]. A user's query to the system is first analyzed by the *User Query Manager *and then decomposed into a combination of acyclic graph and regular graph queries, which are handled by their respective query engines (Figure 1). The system uses two graph query engines to execute specialized algorithms [30] customized for each kind of graph. Both engines access the stored data and indices through an API exposed by the *Warehouse Manager *that provides logical access to the stored data and indices.

In contrast to the visual integration systems such as Cytoscape and VisANT, PathSys has a more comprehensive data model such that the semantic concepts of biological objects, molecular states, and interaction types are more closely mapped to the data elements as shown in Figure 2. The former visual integration systems have a client-end graph manipulation engine with some basic operations, and most data manipulation operations are performed through plug-in function modules. These, however, do not have a server-side graph and relational query engine that can evaluate and optimize arbitrary combinations of operations in a scalable fashion. While the BiologicalNetworks interface does allow a subset of these operations, the full power of the PathSys engine is accessible through the query language described in a later section.

### The PathSys data model

A number of systems such as Cytoscape models MIGs as a ternary relation (node1, edge-label, node2), where the edge-label specifies the nature of interaction. We find that model to be inadequate for the following reasons:

(1) Nodes should not only represent proteins or genes, but should also designate their state while participating in an interaction.

(2) For complex molecules, one needs to distinguish between the interactions of the complex and those of the component molecules.

(3) Mechanism should be available to add as many interaction properties as needed and capture more abstract types than is possible with simple labeled edge.

(4) One needs to represent the fact that one interaction can be regulated by the occurrence of other interactions, thus necessitating a (hyper-)edge that connects two (or more) other edges.

In PathSys we distinguish three types of nodes: *primary node*, *connector node *and *graph node*.

#### Primary node

All macromolecules (e.g. DNAs, RNAs and proteins), small molecules (e.g. ions, ATP, lipids) and physical events (heat, radiation, mechanical stress) are under 'primary node' definition.

#### Connector node

**Figure 3:**
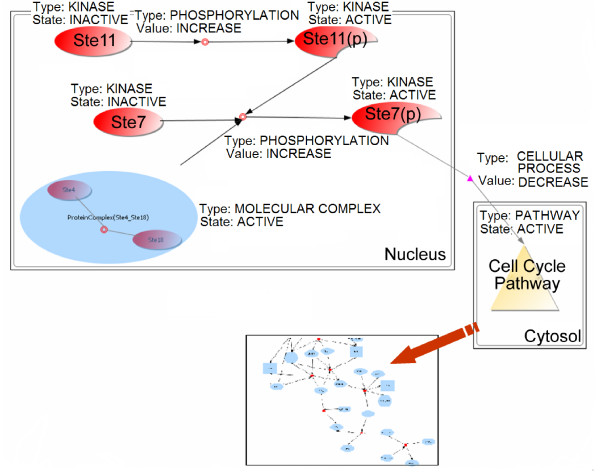
Representation of a simplified model of mating pheromone activated signal transduction pathway.

A connector node is designed to depict the properties of a relationship between a set of source nodes and a set of target nodes. All types of interactions (binding, chemical reaction, expression, etc.) are represented by connector nodes. Note that a connector node is not a simple edge label but a placeholder for "interaction type" and "interaction properties", as shown in Figure 3. The interactions as we stated are m:n relations. Hence we can represent interactions such as chemical reactions with m reactants and n products. The reason for implementing edges as connector nodes with their own properties is that an integration system should be designed to be extensible to hold different information coming from multiple sources. If we have two sources describing a protein-DNA interaction between a protein-node P and a "chromosome-fragment" node D, it is quite possible that these two sources will specify two different properties about this interaction. For example, one source could state that the interaction is that of "transcription factor binding" while another source might state that this interaction is conserved in other species. Modeling the connectors as special nodes allows us to seamlessly scale up by adding as many node properties as needed as information on that edge grows. This could not be accomplished if interactions were modeled just as labeled edges. We illustrate the role of a connector node in terms of the expressive power of the system. Consider the edge as a triple (n1 'activates' n2), where n1, n2 are node constants and 'activates' is an edge name (i.e., an edge label). Our query system allows us to associate a variable x to the edge, thus representing it as x: (n1 'activates' n2). Now the triple (n3 'inhibits' x) is equivalent to the statement "n3 inhibits the activation of n2 by n1". Graphically, this would be represented as an "edge" between the node n3 to the connector node between n1 and n2. Now we can construct queries like "Find all proteins which have properties P1 and P2 and regulate the activation of n2". The answer will find n3 (if n3 has P1 and P2). Similarly, we can represent "competing" interactions as x: (n1 'activates' n2), y: (n3 'activates' n4), (x 'competes_with' y), where the last clause is an "edge" between a pair of connector nodes.

#### Graph node (Hypernode)

In biological systems molecules often form clusters and groups for performing tasks, behaving like a single state. In our system all complex objects (protein complexes, cellular processes) that might contain graphs are defined by graph nodes (hypernodes) (VisANT [26,27]. Binding relations within the hypernode are presented as well. A molecular complex like the *proteasome *is treated as a hypernode, of the type molecular complex. The hypernode gets its own node identifier that is distinct from all nodes (proteins that form subunits of the proteasome). A hypernode may have interactions with single nodes or other hypernodes in the graph. Moreover, members of the hypernode can independently participate in different processes. A hypernode may contain members from different cellular compartments. These features are incorporated in the notion of *Graph Node*. For visual representation of metanodes see Additional file or supplemental materials at [[46], Section: Data Visualization].

Hypernodes play a crucial role in processing graph queries such as path and neighborhood finding, the algorithmic details of the use of hypernodes in query evaluation are provided in supplementary materials.

The internal data model of the graph (Figure 2) consists of a node type hierarchy N ('child of' relation in the NodeType view), an attribute category hierarchy A ('child of' relation in the AttributeTypeCategory view), bags of nodes N and edges E and a data source D.

For some node types, *e.g*. gene, one can specify rules to automatically create *derived node types *such as *mRNA(gene) *and *protein(gene)*. The node type hierarchy N can be a directed acyclic graph because it admits multiple inheritance; for example, *an nuclear transcription factor *is both an *nuclear-localized protein *and a *transcription factor protein*.

We distinguish between the type of the attribute, which reflects its storage data type, which might be the tuple {int, int} for a specific case, from its *semantic category *which might be a "chromosomal interval". In our model, attributes are attached to node instances rather than node types. Thus, if one source provides one set of attributes for a node and a second source provides a different set of attributes for the same node, we can combine both sets of attributes. This enables us, for example, to unite putative transcription factor binding sites from Yeast Promoter database from Cold Spring Harbor Labs and intergenic binding probability information from MIT data [24] on compatible chromosomal intervals.

To illustrate our graph model, consider the highly simplified fact that activation of Ste11 to the phosphorylated state Ste11(p) increases the rate of phosphorylation of another protein Ste7 that is thereby activated (Figure 3). Simultaneously, the molecular complex of Ste4 and Ste18 proteins also increases Ste7 phosphorylation. Activated Ste7 ultimately inhibits the process of cell cycle by producing a G1 mitotic checkpoint arrest [31]. The nodes in this case are Ste11, Ste7, Ste4, Ste18, Ste11(p) (phosphorylated), Ste7(p) of *protein *type and *kinase *subtype; two Graph Nodes: protein complex and cell cycle pathway; and Connector Nodes: two nodes of type *phosphorylation*, and one node of type *Cellular Process*. An edge incident to a connector node denotes that the source nodes participate in the process depicted by the connector node. An edge from a connector node denotes that the process represented by the connector node impacts the target nodes of the edge. The choice of using the connector node implies that the so-called edge label is now a property of the connector node. Syntactic sugar in the query language can specify a query in terms of the *edge label*, and the system translates it to a query on the connection nodes. Defining a few special edge types can connect two primary nodes without having to go through a connector node. We describe two such special edge types here. The first is a *subgraph *edge (edge.relationship = 'subgraph') – it goes from a graph type node to another graph type node where the latter is a subgraph of the former, which, for example, can create named subgraphs. A subgraph may be named (i.e. assigned a separate id) for semantic reasons; for instance, it represents a functional subgroup of interacting proteins within a larger interaction graph. Alternately, a subgraph is named because it has a special property. For example, the system indexes all cliques with more than 3 members. These cliques are denoted as special graph nodes that are used during query processing. A second special edge is a *member-of *edge between a node n and a graph-typed node g that designates that n belongs to the graph represented by g.

#### Graph attributes

A significant class of systems biology queries addresses graph-theoretic properties of source graphs as well as the integrated graph. PathSys maintains a set of graph attributes for each source graph to answer these aggregate queries. At present they include in and out degrees, betweenness centrality and clustering coefficient. Centrality is defined as **b_k _= **∑_ij _(g_ijk_/g_ij_), where g_ij _is the number of shortest paths from node *i *to node j, and g_ijk _is the number of shortest path from *i *to *j *that pass through *k*. For node k, clustering coefficient is the ratio of the number of *k*'s edges to the maximum number of possible edges between *k*'s neighbors. These parameters, together with other measures, such as the graph diameters, are maintained and indexed using conventional index structures. For regions of the graph where neighboring nodes have high clustering coefficient, a "clustering coefficient" attribute is maintained by creating a system-defined graph node that represents the highly connected neighbors. Inclusion of any number of such attributes is possible.

### Integrating graph sources

The task of integrating a new data source to an existing integrated graph schema consists of three steps – defining a new, unpopulated data source in the integrator, mapping the just-imported schema to nodes, node attributes, and edges of the integrated graph, and expressing conflict resolution policies.

#### Source definition

An external data source can be a relational database schema, a tree-structured XML document, an RDF-styled triplet that describes an edge set of a graph, or a DAG structured OWL [32] document. Typically, a new ontology or a node/attribute type hierarchy, such as the phenotype classification tree from MIPS, is presented to the system using a tree (here as an OWL description) data, and a collection of node/edge instances and node properties are presented as relational data. To import this data into PathSys, we first define a new data source

   CREATE DATA SOURCE yeast phenotype (

   fullname 'Yeast Phenotype Classification',

   reference localhost://phenotype.owl',

   description...)

   format XML-RDF-OWL;

where the newly imported data is nicknamed yeast phenotype. XML-RDF-OWL is a format known to the system. For a relational data source, we would declare the format as SQL. With the data source defined, now we specify a PathSys schema element for the new source.

   CREATE TREE phenotype tree (

   version STRING VALUE '2.3',. . .)

   SOURCE yeast phenotype;

#### Schema mapping

The task of schema mapping is to specify how an element of the imported source should be interpreted as an element of the internal schema of PathSys. In PathSys a tree is a special case of graph that is internally used for query evaluation. In a tree structure source, the OWL schema populates the node type hierarchy in Figure 2. The *mapping declarations *are:

   IMPORT NODE TYPE FROM yeast phenotype (

   Class as name,

   )GRAPH phenotype tree

   IMPORT RELATIONSHIP FROM yeast phenotype(

   subClassOf as child of

   )GRAPH phenotype tree

In relational mapping the source integration imports a relational schema (a fragment of the MIPS database) into the graph elements of the internal model (see supplemental material for detail). For each schema mapping, the wrapper generator automatically creates the code to populate the PathSys schema from the new data source.

Once the new graph is integrated, the system computes all graph indices for the new incoming graph and updates indices for the whole integrated graph. Detailed information on how the data are physically represented and the Data Definition Language are provided in Additional file or supplemental materials at [[46], Section: Architecture].

#### Conflict resolution

Crucial to information integration process is resolution of data conflicts. Reconciliation problems are detected by a set of conflict detection rules and are resolved by expert user intervention. Here are some example rules:

(1) Two genes with the different names have the same chromosomal location. For this, we have an automated reconciliation procedure assigning multiple names as synonyms to the same ORF.

(2) Two genes with the same name have different chromosomal location. Problems like this are due to different assigning of gene boundaries, alternative splicing etc. and are resolved by scientists.

(3) Several genes have names such that one name is contained in the other, e.g., 'IME1', 'IME1-TAP(342–531)' and 'IME1(modified:Thr:210)'. The first record refers to the gene IME1, the second to a fragment of gene IME1 that is modified by fusion to a domain called TAP, and the third to the protein encoded by IME1 (IME1p) with the qualifier that the amino acid 'Thr' at the 210-th position was modified. Thus, the records seemingly referring to an item called 'IME1' really refer to objects that are not equal and must be resolved by an expert.

(4) Two genes with different names and chromosomal locations have over 95% similar graph neighborhoods. Products of such genes are likely to be part of the same protein complex and/or have physical interaction. Cases like this can be the starting points for biological discovery to identify functionally related candidate genes.

### Querying graphs in PathSys

*BioNetSQL*, our query language for interaction networks, has the flavor of SQL that can be queried on sets and bags of nodes, edges and their attributes, but additionally allows the returned values to be bags of paths, trees and graphs. Further, the language allows path, tree and graph operations. While a complete description of the language and the query evaluation process is beyond the scope of this paper, we present a few features of the language through one example where we use graph operations in the body of the query and the return data type is a graph. "Find networks of co-localized proteins that are parts of protein complex and are connected by either a 2-hybrid (y2h) edge or a coimmunoprecipitation (coIP) edge."

   SELECT

   graph(N2(n.name, n.source),

   E2(e.label, e.source))

   FROM

   yeastGraphDBG1(N, E)

   WHERE

   n:N and c:N and e:E

   and n.type << 'protein'

   and c.type = 'protein complex'

   and (e.label = 'y2h' or e.label = 'coIP')

   and pathExpr(G1, c// [member of]n) = true

The query declares a variable c whose type is protein complex. The query returns a graph whose nodes n should be tuples with the attributes name and source (i.e., data source), and whose edges e has a label and a source from which that edge is known. Recall that the system will convert this to a query on a connector node. The << operation specifies that the type of the node is "under" "protein" in the node type hierarchy N. The last line reads as "n has an edge whose label has the value member, and this edge points to c", where c is declared above. Note that we did not mention the relationship between nodes n and edges e, namely, an instance of the returned edge set e connects instances of the returned node set n. This constraint, expressed as n.edge = e, is implied by the construct of line 2, where n and e are constrained to be parts of the same graph. For more features of the language and examples see supplemental material.

## Discussion

We developed PathSys to address the limitations of using information from single databases for biological discovery.

Using the high throughput query abilities of PathSys and custom-designed queries for data retrieval (for detailed description of the experiments: filtering procedures, sample queries and results, statistics see Case Study section of Addition file or supplementary materials at [50]), we constructed a global, high confidence network of protein-protein interaction in *S. cerevisiae*. Any of the sub-networks or modules from this comprehensive network can further be extracted and extended to include DNA-protein interactions and genetic interactions to gain more insights into transcriptional regulation of interacting proteins as demonstrated by the MAPK and cell cycle queries outlined below. Additional applications of network queries include expansion to low-confidence interactions for hypothesis generation, network topology studies, deriving regulatory networks for dynamic modeling etc.

### Construction of high confidence integrated network

A challenge in using high-throughput data is selecting high confidence information. We used the strategy of re-enforced edges to minimize error propagation. Using a set of graph queries we performed the following. 1) Protein-protein interactions from MIPS were filtered to remove high-throughput (HTP) interactions contributed by yeast two-hybrid (*y2h*) and co-immunoprecipitation (*co-IP*) studies to construct MIPS_HC (1207 nodes, 1785 edges). 2) To get high confidence interactions (HTP_HC_all) we took the union of two *y2h *data sets [23,33] and its intersection with union of two *co-IP *data sets [34,35], using matrix interpretation for *co-IP *data. Intersection was taken to enhance credibility of true positives over false positives. 3) High confidence DNA-protein network (MIT_HC, 2420 nodes, 4365 interactions) was constructed from Lee *et al*. [24] data filtered for a P-value threshold of 0.001. 4) Genetic interactions from MIPs and Tong *et al*. [7,8] were added to the high confidence DNA-protein interaction data and all interactions from this data set that were supported by at least one high throughput protein-protein interaction evidence were used to construct genetic_HC (289 nodes, 490 interactions). 5) A high confidence, integrated interaction network (All_HC) was derived by taking the union of MIPS_HC, HTP_HC all and genetic HC (1469 nodes, 2997 interactions, connected component of 1037 nodes). The strategy to either combine or intersect the various datasets was determined depending upon the propensity of false positives and false negatives in individual datasets, always aiming for maximum coverage across the genome. This network (see summary Venn diagram of the data in supplementary materials) is a potential first goal for a user interested in a more specific biological process. A more comprehensive network (FYI_HMI) incorporating MIPS complexes and computational predictions [36] for reinforcement as well as retrieval of Cell cycle network is provided in Supplementary materials. In all these applications an important impact of the "information integration" is to place different forms of interactions (such as physical interactions, and different forms of direct and indirect genetic interactions) between proteins and their transcribing genes on the same combined graph. This does not necessarily mean that the physical interactions imply the genetic interaction or vice versa. It simply represents a comprehensive picture of what is known about the neighbourhood of a pair of genes, from which a scientist might develop a hypothesis based on the integrated information.

### Retrieving complex interaction network

To compare the organizational structure of primary protein interaction network to that of a higher order network of organized protein complexes (hypernodes), we derived a network of high confidence protein complexes from MIPS that are directly linked to each other via high-confidence protein-protein interactions (see figure in Supplemental materials). In this network with 164 nodes and 482 interactions, each node represents a protein complex identified by a complex_ID label from MIPS and edges are inter-complex protein-protein interactions from high-confidence HMI network. We analyzed this network for betweenness centrality (BC) to see which of these complexes are potential connection hubs providing shortest paths for communication between different complexes representing various functional modules. The motivation arises from the finding that high BC proteins are functionally significant [37]. Here we address the centrality of protein complexes rather than individual proteins. Complexes with fifteen highest BC values and their functional annotations are shown in Table #1 in Supplemental materials. As expected, majority of the high BC nodes include protein complexes forming cytoskeletal structural elements (actin, tubulin, spindle pole body) as well as complexes involved in general regulatory mechanisms such as SAGA complex, SRB mediator complex and RNA polII complex.

### Integration generates new knowledge

**Figure 4:**
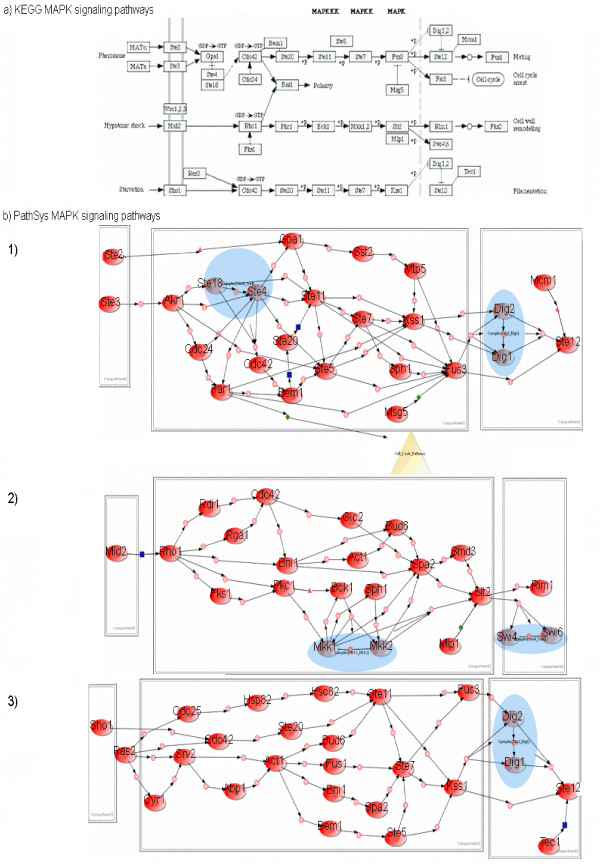
**MAPK signaling pathways produced by PathSys in comparison with the canonical KEGG MAPK signaling pathways. **a). MAPK signaling pathways in Yeast reproduced by KEGG.b). Pathways generated by PathSys for (b1) Pheromone response, (b2) Cell Wall modeling and (b3) Filamentation of MAPK signaling pathways, with the proteins as red ovals, complexes as blue ovals; processes (binary and multiple) and interaction types as small colored circles, squares, diamonds, *etc*.; pathways (Cell Cycle pathway) as yellow triangles, compartments as grey boxes. Network graphs are produced by **BiologicalNetworks **client software.

An excellent benchmark against which to validate our approach is MAPK pathways involved in pheromone response, filamentous growth, and maintenance cell wall integrity (Figure 4), one of the most thoroughly studied networks in yeast, conserved across all eukaryotes. The pathways are activated by G protein-coupled receptors and characterized by a core cascade of MAP kinases that activate each other through sequential binding and phosphorylation reactions.

Two sub-networks were constructed from the MIPS HC as well as ALL_HC networks by selecting for genes whose names begin with STE* and their immediate neighbors. The sub-network derived from MIPS (MAPK_MIPS) shows 37 genes and 74 interactions where as the sub-network from ALL_HC (MAPK_allhc) shows 39 genes and 106 interactions (for the whole network of MAPK neighborhood and intermediate networks see Additional file or supplemental materials at [[46], Section: Case Studies]).

### Validation and insights from integration

To show the impact of MIG integration in understanding biology, we present a comparison between our results and those obtained from KEGG. First, we start with ALL_HC and extract a subnetwork of genes related to "pheromone response" (Figure 4b1). Compared to KEGG (Figure 4a), our results include more members of heterotrimetric G protein complex, including the alpha, beta, and gamma subunits, the GDP-GTP exchange factor, and the GTPase-activating protein (Gpa1p, Ste4p, Ste18p, Cdc24p, and Sst2p, respectively). The Sst2p does not appear in KEGG MAPK pathways. Our model also includes Far1p, a protein necessary for pheromone-induced cell cycle arrest in G1 [38], Mpt5p, a protein necessary for recovery from cell cycle arrest [39], and Bem1p and Sph1p, both of which are necessary for establishment of cell polarity during budding [40,41]. Neither Mpt5p, nor Sph1p appears in the KEGG MAPK pathway. In addition to direct interaction between pheromone receptor (Ste2p and Ste3p) and heterotrimetric G protein complex (Ste4p/Ste18p/Gpa1p), our result contains the interaction of Akr1p, a known inhibitor of signaling in the pheromone pathway [42], with the G protein complex, a fact missed in KEGG MAPK pathways for pheromone response.

Figure 4b2 shows the result of extracting network for the function "cell wall remodeling" from ALL_HC. It contains both GTPase constituents, Rho1 and Cdc42p, as well as associated GAPs and other interactors, including Rdi1p, Rga1p, and Gic2p. The last three proteins are not presented by KEGG (Figure 4a). Fks1p syntase, the actin protein Act1p, and the proteins Bni1p, Bud6p, and Sph1p, which are associated with Rho-mediated signal transduction, actin filament organization, cell polarity establishment, and bud growth are also included. KEGG cell wall integrity pathway misses some of these actors. Membrane proteins Wsc1p, Wsc2p, Wsc3p or Mid2p may fail to interact when forced into the nucleus by the requirements of the standard two-hybrid technique and none of them was encountered in our network.

In the result of a query for the filamentation pathway (Figure 4b3), key components of the Ras GTPase are included, such as Cdc25p (the Ras guanine nucleotide factor), Cyr1p (the Ras-associated adenylate cyclase), and Srv2p, which enables the activation of adenylate cyclase by Ras2p. Several proteins with roles in actin filament organization, cell polarity establishment, bud growth, and GT-Pase mediated signal transduction are shared with the cell wall integrity pathway, including Bni1p, Spa2p, Bud6p and Act1p. The model shows interactions between Abp1p and both Srv2p and Act1p, consistent with the function of Abp1 in tethering Srv2p to cytoskeleton. The adenylate cyclase and associated proteins mentioned above, along with Hsp82p and Hsc82p, activate the cAMP pathway [43], a pathway that acts in parallel with the MAPK pathway to promote filamentation. Hsp82p is a chaperon protein required for activation of the pheromone signaling pathway components [44], and for the general response to amino acid starvation [45]. Most of the facts of the filamentation pathway described above are missing in the KEGG MAPK filamentation pathway.

The MAPK neighbourhood study shows that in spite of KEGG's ontology, our data integration produces more relationships, and thus lends more scientific insight that are not obtainable otherwise.

**Figure 5:**
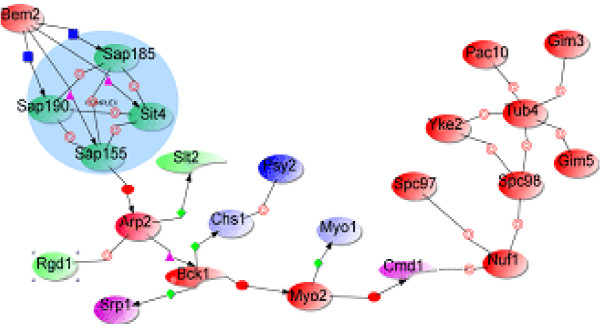
A functional module (largest connected component) for cytoskeletal organization and coordination. Edge line types represent interaction types and node colors represent GO annotation.

Recall that we started the above queries by first creating a high-confidence network (a view over the integrated data). If we start with a less stringent network by including less strongly supported edges, we can use the system as a *hypothesis generator*. For this, we start with a query to extract the Genetic_HC network (described under network construction) representing pairs of genes/proteins that have at least one evidence of protein-protein interaction and at least one evidence of either genetic or DNA-protein interaction has 16 connected components of 5 or more nodes. The next query filters out the largest connected component (Figure 5), which contains 23 nodes, representing functionally related genes/proteins related to cytoskeletal element organization and coordination of cellular function: from the left hand side, protein folding pathway components (Pac10, Gim3 and Gim5) interact cytoskeletal element nucleating proteins (Tub4 and Spc97) to nucleate and assemble (Yre2) microtubule and actin filaments, which interface through Spc98 with a spindle pole body component (Nuf1) during mitosis, which in turn interacts with calcium regulated signaling pathway through the calmodulin Cmd1, and thus regulates organelle movement (through Myo2) and cytokinesis (through Myo1) during cell division. From the right hand side, the Rho-GTPase activating protein (Rho-GAP) Bem2, acting in a multi-protein complex with Sit4, Sap185, Sap190, and Sap155, transduces a signal from the cell wall to the cytoskeletal elements through Arp2 and Rgd1 (another rho-GAP), and thus to the specialized MAPK signaling pathway (through Bck1) involved in cytoskeletal reorganization. We thus hypothesize that these interactions trigger cell wall repair and morphogenesis through Chs1. The derivation of this set of genes/proteins as a recognizable modular unit for cellular organization, without making any explicit query related to cytoskeletal elements, is the first of its kind and depended critically on the ability to integrate multiple databases.

### Statistical properties of integrated networks

Another important area of current research is beginning to address how molecular networks having different functional significance but comprised of the same elements evolve.

Addressing such questions is possible only when data sets with very different types of data are integrated. To be most useful integrated networks should be constructed by queries, and statistical tests conducted by queries through algorithms established over the database. As an example, we studied three high-confidence sub-networks (protein-protein [36], DNA-protein MIT_HC, genetic MIPS_HC) in yeast in a pair-wise manner. In these networks, a node is a gene or a protein, and the conditions specified are expressed as queries over the integrated network. In queries for each functional network above, we issued aggregate queries to compute the degree, the clustering coefficient and the betweenness centrality of the nodes respectively. We examined for each common node whether a particular topological property shows statistical inter-dependence between any two networks. For each comparison, we used two statistical tests for inter-dependence: a correlation test and a χ^2 ^test to determine whether there are systematic rules of association in the three networks that govern the allowed interaction topologies of individual network members across the different functional networks. Results of this study will appear elsewhere (Raval *et al*. in preparation). Note that it is possible to perform analysis like this automatically mainly due to the graph-based integration of molecular interaction from different sources, even though the individual data sources had very detailed data content.

## Conclusion

The approach we have presented facilitates graph information integration from multiple sources and allows one to query and retrieve biologically interesting relations between genes and proteins, and obtain topological properties on integrated graphs for biological hypothesis testing. The system, implemented on top of Oracle DBMS, uses a novel graph query language and evaluation engine to process complex queries some of which are illustrated here. It is now possible to interrogate simultaneously and at several levels of detail complex interactomes to return networks of interactions with multiple semantic features.

We showed that our integration approach is able to provide biologically interesting information not possible with existing databases. For example although the pheromone response pathway is commonly depicted as a linear transmission of the mating signal from the membrane receptor to the nuclear effectors via a MAPK cascade, the real picture of cellular processes and interactions is not that simple; the topology of interactions is considerably more complicated than a series of pairwise interactions. This is captured well through our MAPK example query. For biological hypothesis generation, we have shown an example of how to retrieve networks of lower confidence but higher biological discovery potential. Finally, multiple integrated networks can be mined simultaneously for graph-properties that encode systems-level information on biological entities, such as molecular-complex integration networks. Thus, data integration and query analysis systems such as PathSys, should become integral tools for future efforts to build a model of a cell as a whole.

## Availability and requirements

**Project name: **PathSys. The PathSys's client software, named BiologicalNetworks, is available as a Java Web Start application at  (for description see Additional file 2). Download version is also available.

Project home page:

 and



**Operating system(s): **Windows 2000 and XP, Linux.

**Programming language: **Java.

**License: **Free for academic purposes.

**Other requirements: **Java 1.4 or higher, not yet available for MacOS.

Any restrictions to use by non-academics: contact the authors.

## Authors' contributions

MB, XQ and AG contributed to system concept. MB implemented the system and performed major programming work. MB, XQ, SG and AlR contributed to data analysis. This work was coordinated by AG and AnR.

## Supplementary Material

Additional File 2 Word documented supplementary material containing description of the PathSys's client called BiologicaNetworks. Click here for file

Additional File 1 Word documented supplementary material containing all additional information and complete description of the PathSys system. Click here for file
