# Supplementary material for: PathSys: integrating molecular interaction graphs for systems biology
Source: BMC Bioinformatics. 2006 Feb 7;7:55. doi: 10.1186/1471-2105-7-55 (PMC1409799; doi:10.1186/1471-2105-7-55)
Supplement: Additional File 2 — Word documented supplementary material containing description of the PathSys's client called BiologicaNetworks. [file 1471-2105-7-55-S2.doc]

## BiologicalNetworks Software

**BiologicalNetworks** is a Systems Biology software platform for biological pathways analysis, querying and visualization of gene regulation and protein interaction networks, metabolic and signaling pathways. It is equipped with filtering and visualization tools, to provide high quality, easily understood scientific presentation of your pathway analysis results.

*BiologicalNetworks* includes [**PathSys**](http://brak.sdsc.edu/pub/BiologicalNetworks/PathSys), general-purpose scalable warehouse of biological information, which integrates over 20 curated and publicly contributed data sources, biological experimental and PubMed data for the Budding Yeast (S. cerevisiae) and Fly (D.melanogaster) . BiologicalNetworks is also supported with curated pathways from a number of public databases like **KEGG** and different scientific studies.

*BiologicalNetworks* identifies relationships among genes, proteins, small molecules and other cellular objects, and draws pathways, linked to the original sources of information.

*BiologicalNetworks* is equipped with enhanced graph manipulation and a query language, data mining and filtering tools, a storage mechanism and a generic data-importing mechanism through schema-mapping.

[
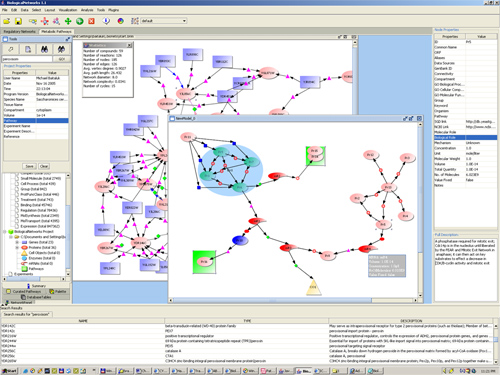
](http://brak.sdsc.edu/pub/BiologicalNetworks/images/2big.jpg)

**Pathways Analysis**

- Import and export your data
- Create, save, edit your pathways and produce high quality diagrams for your publications
- *Meta-Network* (network in which a node, *Meta-Node*, itself has its internal network structure): multi-scale visualization of bio-networks, ideal for network of functional modules.
- Find relationships among genes/proteins, cell processes and other objects
- Optimize the view by filtering, pathway expansion, and protein classification.
- Perform graphic drawing and layout optimization

**Navigation and Tools**

- Build Molecular Interaction Networks for gene lists imported from microarray and other experiments
- Specify and visualize upstream and downstream events
- Find a path between two or several molecules
- Detect common targets or/and regulators for a group of proteins.

**Pathways and Interaction Networks from public Databases and Literature**

- Browse PathSys pathway database system compiled from over 20 databases
- Access more than 140,000 facts of regulation, interaction and modification
- Get the original sentence or paper abstract to validate the facts of interaction and biological phenomenon.

BiologicalNetworks pathways analysis software supports Windows® 2000/XP and Linux operating systems.
