# Supplementary material for: PathSys: integrating molecular interaction graphs for systems biology
Source: BMC Bioinformatics. 2006 Feb 7;7:55. doi: 10.1186/1471-2105-7-55 (PMC1409799; doi:10.1186/1471-2105-7-55)
Supplement: Additional File 1 — Word documented supplementary material containing all additional information and complete description of the PathSys system. [file 1471-2105-7-55-S1.doc]

## The PathSys System

**PathSys is a graph-based system for creating a combined database of biological pathways, gene regulatory networks and protein interaction maps. In PathSys are integrated over 14 curated and publicly contributed data sources for the budding yeast (S. cerevisiae), as well as Gene Ontology, which is structured as an acyclic graph.**

The data sources are:

- Co-immunoprecipitation data (Gavin, A. C. et al. (2002) "Functional organization of the yeast proteome by systematic analysis of protein complexes". Nature, 415, 141–147.)
- Co-immunoprecipitation data (Ho, Y. et al. (2002) "Systematic identification of protein complexes in Saccharomyces cerevisiae by mass spectrometry". Nature, 415, 180–183.)
- Yeast-two hybrid data (Ito, T. et al. (2001) "A comprehensive two-hybrid analysis to explore the yeast protein interactome". Proc Natl Acad Sci U S A. 98, 4569-74 )
- Yeast-two hybrid data (Uetz, P. et al. (2000) "A comprehensive analysis of protein-protein interactions in Saccharomyces cerevisiae". Nature 403,623-7.)
- Genetic interaction data (Tong, A. H. Y. et al. (2001) "Systematic genetic analysis with ordered arrays of yeast deletion mutants". Science, 294, 2364–2368.)
- Genetic interaction data (Tong, A.H. et al. "Global mapping of the yeast genetic interaction network". Science 303, 808-13 (2004).)
- MIT data <http://web.wi.mit.edu/young/regulatory_network/>, (Lee, T. et al. (2002) "Transcriptional regulatory networks in Saccharomyces cerevisiae". Science, 298, 799–804.)
- UCSF localization data (Huh, W.K. et al. (2003), "Global analysis of protein localization in budding yeast". Nature 425,686-91.)
- MIPS data (<http://mips.gsf.de/genre/proj/yeast/>)
- CSH data (<http://cgsigma.cshl.org/jian/>)
- Transfac (<http://www.gene-regulation.com/>)
- BIND database (<http://bind.ca/>)
- pre-BIND database (<http://www.blueprint.org/products/prebind/>)
- KEGG (<http://www.genome.ad.jp/kegg>)

**Introduction**

1. PathSys System Architecture
2. Database Schema
3. Representation of the Data
4. Physical Storage Schema (DDL)

### PathSyS System Architecture

PathSys system consists of six major parts: Client Side Application, Graph Query Engines, Integration Client/Manager, Data Importer, Schema Mapping Tools and Data Warehouse.

Client Application implements all business logic and a significant part of the user interface. Two novel Graph Query Engines store and query molecular interaction network and directed acyclic graphs such as ontologies and taxonomies, using specialized algorithms (see Chen et al. (2005) for example) customized for each kind of graph. Data Importer can accept data from the external data source, validate it against the schema, and store the data in the warehouse. Integration Client/Manager specifies a new database that needs to be integrated in the system. The user provides the system with the schema of the new source, and the schema is validated and stored in the Schema Library


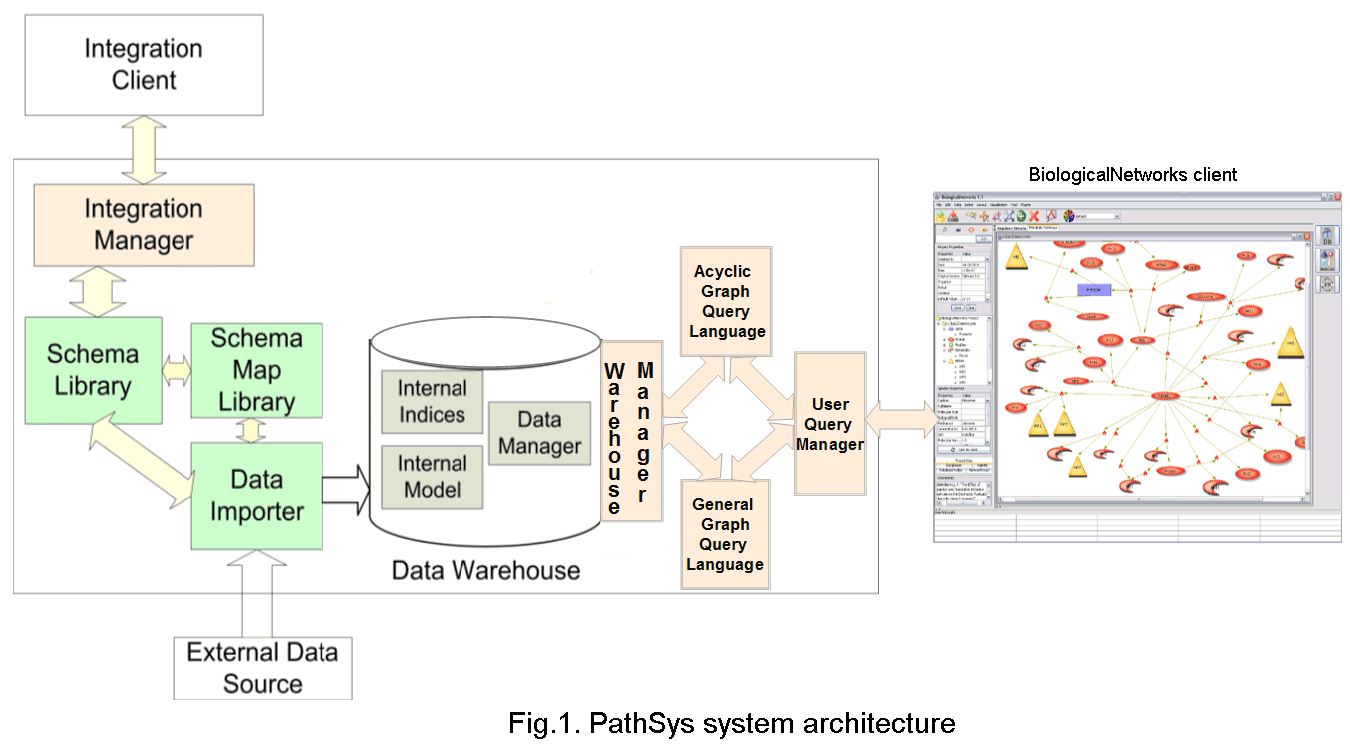


### Database Schema

The PathSys database was designed to represent generic network data.

Current implementation defines three classes of vertices – *primary nodes* (primary objects), *connector nodes* (events of interaction or regulation between primary objects) and *graph nodes* (complex objects (protein complexes, cell processes) that might contain graphs). Connector nodes are identified by mechanism and effect type. Vertices are stored in the table *Nodes*. Nodes themselves can be of several types (Proteins, Small Molecules, Cell Processes, Expression Controls, Binding, Protein Modification etc.), which are recorded in the table *NodeType*.

The current implementation supports three classes of links between vertices– *directed* and *non directed* (as defined by the field Direction in the table Edges) and *membership* (as defined by the field *Relation* in the table *Edges*). Directed links can describe biological notion of regulation, such as “protein A activates protein B”; non-directed links are used to describe binding events: “protein A binds protein B”; membership links describe situations such as “protein complex P contains protein A”. The database structure does not limit the number of different classes of nodes and edges. Also, there can be any number of node types, attribute types.

The two component networks that we promote are a natural extension of the data models accepted for metabolic databases, and will provide a unifying ground for such diverse phenomena as signaling pathways, protein interaction maps, and gene expression networks.

Any node can have an arbitrary number of associated attributes. Node attributes usually store information about functional class, localization, chemical structure etc. These attribute, as well as names of proteins and other objects, are searchable fields. *Attribute* values can be translated into color or shape coding of nodes during visualization. Connector node attributes store information about tissues, cell types, experimental conditions, and other biologically meaningful details. All fields are searchable.

*Attribute* of an object can be assigned a single value, as well as multiple values. Different properties can form sets to describe complex attributes, such as experimental conditions that might include cell type, tissue, organism, species, p_values etc.


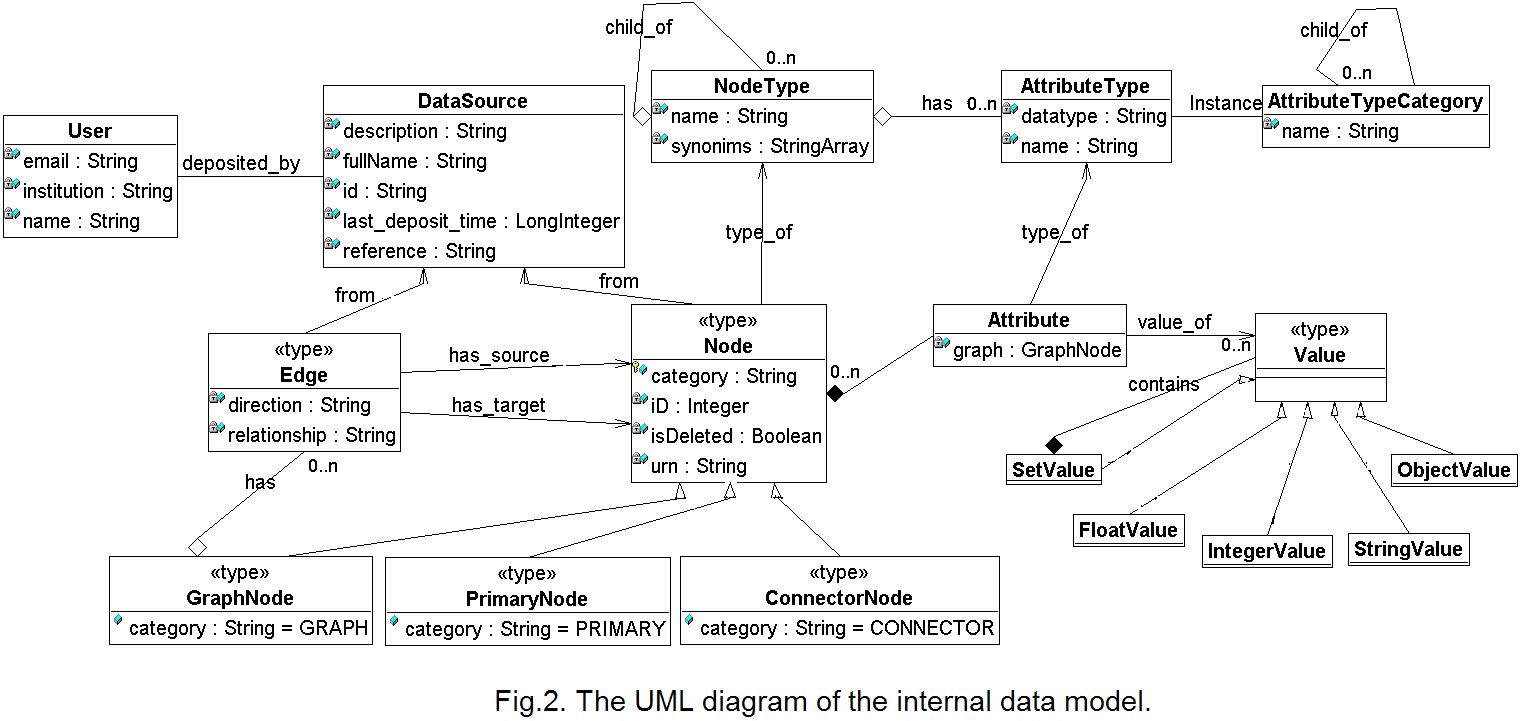


### Representation of the Data

##### Database tables

Nodes - Stores primary, connector and graph nodes

ID - Internal node ID [auto number]

URN - Global node ID [string]

Node_Type- Node type ID (Protein, Cell Process, Binding, etc.) [number]

Node_Class- Node class ID (Node, Control) [number]

isDeleted - “true” if node has been deleted

DataSource- External database source name where the node comes from

Edges - Stores links between nodes (primary, connector and graph nodes)

ID - Internal link ID [auto number]

IDSrc - Internal ID of the source node [number]

IDDst - Internal ID of the destination node [number]

Direction - Link class (directed, non-directed)

Relation - Special relationship types(subgraph, member-of, ...)

Graph_Edges - Stores information about graph edge membership, for performance purposes, generally for graph nodes (complexes) searches

graph_ID - Internal graph ID [auto number]

Edge_ID - Link ID (from Edges table) [number]

Graph_Nodes - Stores information about graph node membership, for performance purposes, generally for

graph nodes (complexes) searches

graph_ID - Internal graph ID [auto number]

Node_ID - Node ID (from Node table) [number]

NodeType - Stores node type information (Protein, Binding, etc.) as hierarchy

ID - Node type ID [auto number]

Name - Node type name [string]

child_of - child of the node type in the node type hierarchy [number]

NodeType_SynonymsID- Node Type synonims ID [number]

NodeType_Synonyms - Stores information about Node Type synonyms

ID - Node type synonym ID [auto number]

NodeType_Synonym - Node Type synonym [number]

Attributes - Stores attributes associated with nodes

ID - Internal attribute value ID [auto number]

NodeID - ID of the corresponding Node [number]

Attribute_Type_ID - Attribute type ID (Name, Organism, Effect, etc.) [number]

AttrOption - Attribute option (multi-value, single value) [number]

DataType - Data type of attribute [string]

DoubleValue - Stores values for dictionary-based properties

ID - Internal value ID [auto number]

Value - Property value

NodeID - Corresponding Node ID. For performance purposes only.

It reduces the number of joins, although the information is duplicated.

IntegerValue - Stores values for dictionary-based properties

ID - Internal value ID [auto number]

Value - Property value

NodeID - Corresponding Node ID. For performance purposes only.

It reduces the number of joins, although the information is duplicated.

FloatValue - Stores values for dictionary-based properties

ID - Internal value ID [auto number]

Value - Property value

NodeID - Corresponding Node ID. For performance purposes only.

It reduces the number of joins, although the information is duplicated.

StringValue - Stores values for dictionary-based properties

ID - Internal value ID [auto number]

Value - Property value

NodeID - Corresponding Node ID. For performance purposes only.

It reduces the number of joins, although the information is duplicated.

LongStingValue - Stores values for dictionary-based properties

ID - Internal value ID [auto number]

Value - Property value

NodeID - Corresponding Node ID. For performance purposes only.

It reduces the number of joins, although the information is duplicated.

Attribute_Type - Stores attribute type information (Name, Organism, Effect, etc.)

ID - Attribute type ID [auto number]

AttributeName - Attribute type name [string]

Attribute_Type_Category - Stores attribute category hierarchy

ID - Internal attribute category ID [auto number]

Attribute_Type_Name- Attribute type name

Relation - Special relationship types (subgraph, member-of, ...)

child_of - child of the node type in the attribute category hierarchy [number]

AT_NT - Stores the attribute type and node type relationships, for performance purposes only

Attribute_Type - Attribute type ID [number]

Node_Type - Node Type ID [number]

Data_Source - Stores the information about different data sources

ID - Internal Data Source ID [auto number]

IDName - Data Source Name [string]

FullName - Full name of data source [string]

Last_Depos_Time - Last time data was deposited

Description - Data source description [string]

Reference - Reference to data source [string]

UserID - Corresponding user's ID [number]

User - Stores the information about different users, allowed to store, update or edit database information

ID - Internal User ID [auto number]

Name - User Name [string]

Institute - User's institution name [string]

email - User's e-mail [string]

### Physical Storage Schema (DDL)

CREATE TABLE NODES

(

ID NUMBER(8) NOT NULL PRIMARY KEY,

URN VARCHAR2(50) NOT NULL,

Node_Type NUMBER(5) NOT NULL,

Node_Class CHAR NOT NULL,

isDeleted boolean,

Data_Source NUMBER(5),

CONSTRAINT Node_Type_FKEY

FOREIGN KEY (Node_Type) REFERENCES NODETYPE (ID),

CONSTRAINT Data_Source_FKEY

FOREIGN KEY (Data_Source) REFERENCES DATA_SOURCE (ID)

);

CREATE INDEX Node_ID_Ind ON NODES(ID);

CREATE INDEX Node_Type_Ind ON NODES(Node_Type);

CREATE INDEX Node_Calss_Ind ON NODES(Node_Class);

CREATE TABLE EDGES

(

ID NUMBER(10) NOT NULL PRIMARY KEY,

IDSrc NUMBER(8) NOT NUL,

IDDst NUMBER(8) NOT NULL,

Direction CHAR NOT NULL,

Relation VARCHAR2(50)

CONSTRAINT IDSrc_FKEY

FOREIGN KEY (IDSrc) REFERENCES NODES (ID),

CONSTRAINT IDDst_FKEY

FOREIGN KEY (IDDst) REFERENCES NODES(ID),

);

CREATE INDEX Edges_Ind ON EDGES(ID);

CREATE INDEX Edges_Ind ON EDGES(IDSrc);

CREATE INDEX Edges_Ind ON EDGES(IDDst);

CREATE INDEX Edges_Ind ON EDGES(Direction);

GRAPH_EDGES

(

graph_ID NUMBER(5) NOT NULL,

Edge_ID NUMBER(10) NOT NULL

CONSTRAINT EDGEID_FKEY

FOREIGN KEY (EDGE_ID) REFERENCES EDGES (ID)

);

CREATE INDEX graph_nodes_Ind1 ON GRAPH_EDGES(graph_ID);

CREATE INDEX graph_edge_Ind1 ON GRAPH_EDGES(Edge_ID);

GRAPH_NODES

(

graph_ID NUMBER(5) NOT NULL,

Node_ID NUMBER(8) NOT NULL

CONSTRAINT NODEID_FKEY

FOREIGN KEY (NODE_ID) REFERENCES NODES (ID)

);

CREATE INDEX graph_nodes_Ind2 ON GRAPH_NODES(graph_ID);

CREATE INDEX graph_edge_Ind2 ON GRAPH_NODES(Node_ID);

CREATE TABLE NODE_TYPE

(

ID NUMBER(5) NOT NULL PRIMARY KEY,

Name VARCHAR2(50) NOT NUL,

child_of NUMBER(5),

NodeType_SynonymsID NUMBER(10)

CONSTRAINT child_of_FKEY2

FOREIGN KEY (child_of) REFERENCES NODE_TYPE (ID),

CONSTRAINT SynonimsID_of_FKEY2

FOREIGN KEY (NodeType_SynonymsID) REFERENCES NodeType_SYNONIMS (ID),

);

CREATE INDEX Edges_Ind ON NODE_TYPE(ID);

CREATE INDEX Edges_Ind ON NODE_TYPE(child_of);

CREATE NodeType_SYNONIMS

(

ID NUMBER(10) NOT NULL,

NodeType_Synonym VARCHAR2(50)

);

CREATE INDEX NodeType_Syn_Ind ON NodeType_SYNONIMS(ID);

CREATE TABLE ATTRIBUTES

(

ID NUMBER(20) NOT NULL PRIMARY KEY,

NodeID NUMBER(8) NOT NULL,

Attribute_Type_ID NUMBER(5) NOT NULL,

attrOption NUMBER(3) NOT NULL,

DataType VARCHAR2(20)

CONSTRAINT NodeID_FKEY

FOREIGN KEY (NodeID) REFERENCES NODES (ID),

CONSTRAINT Attribute_Type_ID_FKEY

FOREIGN KEY (Attribute_Type_ID) REFERENCES ATTRIBUTE_TYPE (ID),

CONSTRAINT Node_Type_ID_FKEY

FOREIGN KEY (Node_Type_ID) REFERENCES NODETYPE (ID),

);

CREATE INDEX Attributes_Ind1 ON ATTRIBUTES(ID);

CREATE INDEX Attributes_Ind2 ON ATTRIBUTES(NodeID);

CREATE TABLE DOUBLEVALUE

(

ID NUMBER(8) NOT NULL PRIMARY KEY,

VALUE NUMBER(10),

NodeID NUMBER(8)

CONSTRAINT DOUBLEVALUE_FKEY

FOREIGN KEY (ID) REFERENCES ATTRIBUTES (ID),

CONSTRAINT NODEID_FKEY

FOREIGN KEY (NodeID) REFERENCES NODES (ID)

);

CREATE INDEX SETVALUE_Ind1 ON DOUBLEVALUE(ID);

CREATE INDEX NODE_Ind1 ON DOUBLEVALUE(NodeID);

CREATE TABLE INTEGERVALUE

(

ID NUMBER(8) NOT NULL PRIMARY KEY,

VALUE NUMBER(5),

NodeID NUMBER(8)

CONSTRAINT INTEGERVALUE_FKEY

FOREIGN KEY (ID) REFERENCES ATTRIBUTES (ID),

CONSTRAINT NODEID_FKEY

FOREIGN KEY (NodeID) REFERENCES NODES (ID)

);

CREATE INDEX INTEGERVALUE_Ind ON INTEGERVALUE(ID);

CREATE INDEX NODE_Ind2 ON INTEGERVALUE(NodeID);

CREATE TABLE FLOATVALUE

(

ID NUMBER(8) NOT NULL PRIMARY KEY,

VALUE NUMBER(8,3),

NodeID NUMBER(8)

CONSTRAINT FLOATVALUE_FKEY

FOREIGN KEY (ID) REFERENCES ATTRIBUTES (ID),

CONSTRAINT NODEID_FKEY

FOREIGN KEY (NodeID) REFERENCES NODES (ID)

);

CREATE INDEX FLOATVALUE_Ind ON FLOATVALUE(ID);

CREATE INDEX NODE_Ind3 ON FLOATVALUE(NodeID);

CREATE TABLE STRINGVALUE

(

ID NUMBER(8) NOT NULL PRIMARY KEY,

VALUE VARCHAR2(50),

NodeID NUMBER(8)

CONSTRAINT STRINGVALUE_FKEY

FOREIGN KEY (ID) REFERENCES ATTRIBUTES (ID),

CONSTRAINT NODEID_FKEY

FOREIGN KEY (NodeID) REFERENCES NODES (ID)

);

CREATE INDEX STRINGVALUE_Ind ON STRINGVALUE(ID);

CREATE INDEX NODE_Ind4 ON STRINGVALUE(NodeID);

CREATE TABLE LONGSTRINGVALUE

(

ID NUMBER(8) NOT NULL PRIMARY KEY,

VALUE VARCHAR2(1000),

NodeID NUMBER(8)

CONSTRAINT LONGSTRINGVALUE_FKEY

FOREIGN KEY (ID) REFERENCES ATTRIBUTES (ID),

CONSTRAINT NODEID_FKEY

FOREIGN KEY (NodeID) REFERENCES NODES (ID)

);

CREATE INDEX LONGSTRINGVALUE_Ind ON STRINGVALUE(ID);

CREATE INDEX NODE_Ind5 ON LONGSTRINGVALUE(NodeID);

CREATE TABLE ATTRIBUTE_TYPE

(

ID NUMBER(5) NOT NULL PRIMARY KEY,

AttributeName VARCHAR2(30) NOT NULL,

CONSTRAINT ATTRIBUTE_TYPE_FKEY

FOREIGN KEY (ID) REFERENCES ATTRIBUTE_TYPE_CATEGORY (ID)

);

CREATE INDEX ATTRIBUTE_TYPE_Ind ON ATTRIBUTE_TYPE(ID);

CREATE TABLE ATTRIBUTE_TYPE_CATEGORY

(

ID NUMBER(5) NOT NULL PRIMARY KEY,

Attrubute_Type_Name VARCHAR2(30) NOT NULL,

Relation VARCHAR2 (50),

child_of NUMBER(5)

CONSTRAINT attrchild_of_FKEY

FOREIGN KEY (child_of) REFERENCES ATTRIBUTE_TYPE_CATEGORY (ID)

);

CREATE INDEX ATTRIBUTE_TYPE_CATEGORY_Ind ON ATTRIBUTE_TYPE_CATEGORY(ID);

CREATE INDEX ATTRIBUTE_TYPE_CATEGORY_child_Ind ON ATTRIBUTE_TYPE_CATEGORY(child_of);

AT_NT

(

ATTRIBUTE_TYPE NUMBER(5) NOT NULL,

NODE_TYPE NUMBER(5) NOT NULL

CONSTRAINT ATTRIBUTE_TYPE_of_FKEY

FOREIGN KEY (ATTRIBUTE_TYPE) REFERENCES ATTRIBUTE_TYPE (ID),

CONSTRAINT NODE_TYPE_of_FKEY

FOREIGN KEY (NODE_TYPE) REFERENCES NODE_TYPE (ID),

);

CREATE INDEX ATTRIBUTE_TYPE__Ind ON AT_NT(ATTRIBUTE_TYPE);

CREATE INDEX NODE_TYPE__Ind ON AT_NT(NODE_TYPE);

CREATE TABLE DATA_SOURCE

(

ID NUMBER(5) NOT NULL PRIMARY KEY,

IDName Varchar2(20) NOT NULL,

FullName varchar2(30) NOT NULL,

Last_Depos_Time Date,

Description varchar2(2000),

Reference varchar2(50),

UserID NUMBER(5),

CONSTRAINT User_ID_FKEY

FOREIGN KEY (User_ID) REFERENCES USER (ID)

);

CREATE TABLE User

(

ID NUMBER(5) NOT NULL PRIMARY KEY,

Name Varchar2(20) NOT NULL,

Institute varchar2(20),

email varchar2(30)

);

## Data Integration

1. Source Definition.
2. Schema Mapping.
3. Sample Schema Mappings.

While the logical model of information in PathSys is graph based, there are in reality a variety of informa- tion sources that provide different components of the integrated graph. A source may contribute only node properties. For example, the Yeast GFP Fusion Localization Database (Huh et al. (2003), Ghaemmaghami et al. (2003)) provides, for each gene or ORF, a set of locations where the gene is expressed. The location is considered a multi-valued property of the gene. A second kind of source contributes graphs. All protein-protein interaction sources like Gavin et al. (2002), for example, may con- tribute an interaction graph from co-immunoprecipitation experiments using a matrix model.

In this case, the type of the edge is physical interaction and the label on the edge includes the type of experiment (co-IP). Yet a third category of sources, may explicitly provide a set of attributes qualifying the nature of the relationship. The protein-DNA interaction data provided by Lee et al. (2002) records a probability value for every interaction between a protein and a DNA region, both for genes and for intergenic regions. Note that this graph is directed while the protein-protein interaction graphs are not. Given this heterogeneity in the type of information that a source may provide, PathSys uses a very generic internal model to accommodate different kinds of sources, such that a new source, providing a new set of nodes, edges, or node/edge properties can be dynamically incorporated into an existing integrated database.

### Source Definition

Currently, the external data source can be:

- a relational database schema
- a tree-structured XML document
- an RDF-styled triplet that describes an edge set of a graph
- a DAG structured OWL (<http://www.w3.org/TR/owl-features>) document.

Typically, a new ontology or a node/attribute type hierarchy, such as the phenotype classification tree from MIPS:


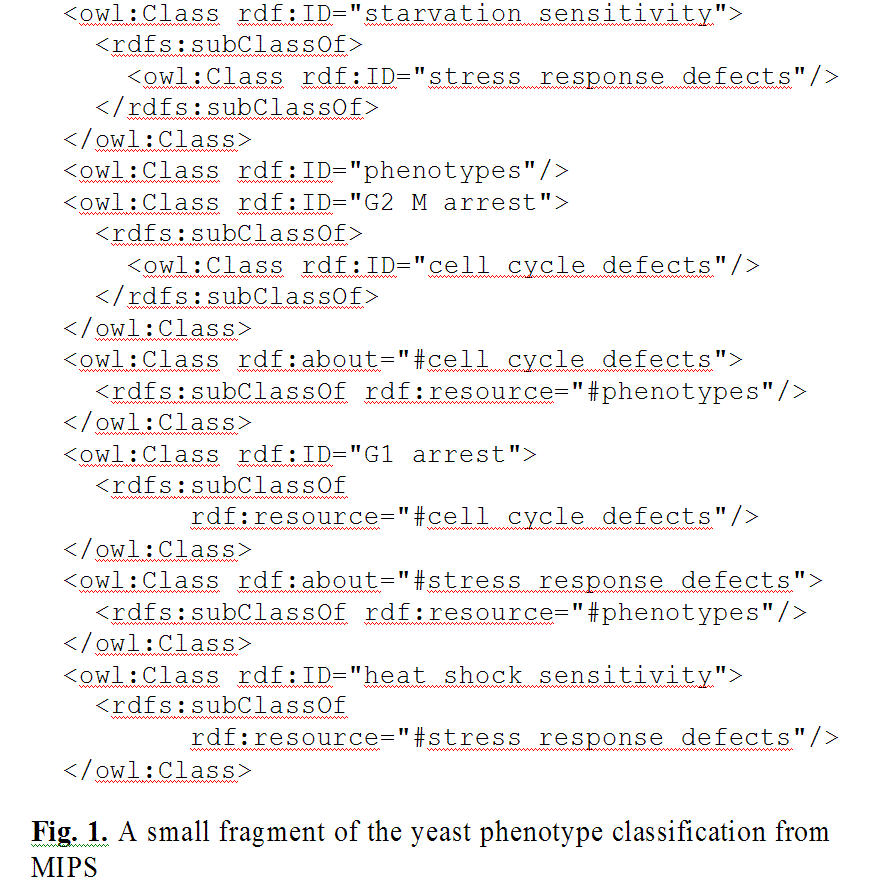


### Schema Mapping

Schema mapping specifies how an element of the imported source should be interpreted as an element of the internal schema of PathSys. We considering the tree-structured and relational data sources. In the first case, we would like the OWL schema to populate the node type hierarchy. The mapping declarations are:

IMPORT NODE TYPE FROM yeast phonotype ( Class as name,

) GRAPH phenotype tree

IMPORT RELATIONSHIP FROM yeast phenotype(

subClassOf as child of

) GRAPH phenotype tree

The second example of source integration imports a relational schema (a fragment of the MIPS database) into the graph elements of the internal model. Figure 2 shows the relational schema. In the MIPS complex relation, the attribute complex references the cid of the MIPS complex category relation. Notice that gene name is explicitly mapped to the pre-existing attribute name. The expression (orf1, gene1) AS SOURCE REFERENCE states that for the edge the source node uses the attribute pair {orf1, gene1} as a foreign key. Also notice how the protein complexes and their members are defined explicitly as hypernodes and hypernode members.

#### Sample Source Schema. MIPS

MIPS_genes (

orf VARCHAR PRIMARY KEY,

gene_name VARCHAR,

coordinates VARCHAR,

classification INTEGER,

description VARCHAR

);

MIPS_interaction (

id INTEGER,

orf1 VARCHAR,

gene1 VARCHAR,

relationship VARCHAR,

orf2 VARCHAR,

gene2 VARCHAR,

description VARCHAR,

reference VARCHAR,

evidence VARCHAR,

FOREIGN KEY (orf1, gene1) REFERENCES MIPS_genes (orf, gene_name),

FOREIGN KEY (orf2, gene2) REFERENCES MIPS_genes (orf, gene_name)

);

MIPS_gene_alias (

orf VARCHAR,

gene VARCHAR,

alias VARCHAR

);

MIPS_complex_category (

cid VARCHAR,

name VARCHAR

);

MIPS_complex (

complex VARCHAR

REFERENCES MIPS_complex_category (cid),

entry VARCHAR,

ref VARCHAR,

evidence VARCHAR

);

#### Mapping directives for MIPS:

CREATE DATA_SOURCE mips

(

fullname ‘MIPS CYGD’,

reference ‘http://mips.gsf.de’,

description ‘The MIPS Comprehensive Yeast Genome Database aims to …’

);

CREATE GRAPH mips_graph

(

version STRING VALUE ‘mips-11-2004’,

… other meta-data …

) SOURCE mips;

IMPORT NODE FROM MIPS_genes

(

orf AS ATTRIBUTE,

gene_name AS ATTRIBUTE name,

coordinates AS ATTRIBUTE,

classification AS ATTRIBUTE,

description AS ATTRIBUTE

)

TYPE gene // node type

GRAPH mips_graph

PRIMARY IDENTIFIER (orf)

SECONDARY IDENTIFIER (gene_name);

IMPORT EDGE FROM MIPS_interaction

(

// skip column ‘id’

(orf1, gene1) AS SOURCE REFERENCE,

(orf2, gene2) AS TARGET REFERENCE,

relationship AS ATTRIBUTE,

description AS ATTRIBUTE,

reference AS ATTRIBUTE,

evidence AS ATTRIBUTE

)

DIRECTION undirected

GRAPH mips_graph;

IMPORT NODE ATTRIBUTE FROM MIPS_gene_alias

(

(orf, gene) AS NODE REFERENCE,

alias AS ATTRIBUTE synonym

) GRAPH mips_graph;

IMPORT HYPERNODE FROM MIPS_complex_category

(

cid AS ATTRIBUTE,

name AS ATTRIBUTE

) GRAPH mips_graph

PRIMARY IDENTIFIER (cid)

SECONDARY IDENTIFIER (name);

IMPORT HYPERNODE MEMBER FROM MIPS_complex

(

complex AS NODE REFERENCE,

entry AS MEMBER NODE REFERENCE,

ref AS MEMBER ATTRIBUTE,

evidence AS MEMBER ATTRIBUTE

) GRAPH mips_graph;

#### Sample Source Schema : Gene Ontology

// two kinds of primary nodes: term and gene_product

Term (

id INTEGER PRIMARY KEY,

name VARCHAR,

term_type VARCHAR,

definition VARCHAR

);

Gene_product (

id INTEGER PRIMARY KEY,

name VARCHAR,

species_id INTEGER REFERENCES SEPECIES(ID)

);

// species table is used to show many-to-one relationship.

Species (

id INTEGER PRIMARY KEY,

lineage VARCHAR,

genus VARCHAR,

species VARCHAR

);

// gene_product_properties is another kind of property table. The property is already

// stored in name-value pair.

Gene_product_properties (

gene_product_id INTEGER REFERENCES gene_product (id),

property_name VARCHAR,

property_value VARCHAR

);

// one-to-many relationship

Gene_product_synonyms (

gene_product_id INTEGER REFERENCES gene_product (id),

synonym VARCHAR

);

// analogous to complex data. A family has one or more members.

// column gene_product_id refers to member’s id.

Gene_product_family (

gene_product_family_name VARCHAR,

gene_product_id INTEGER REFERENCES gene_product (id),

putative BOOLEAN // it is a property on the member

);

// edge table

Term2term (

term1_id INTEGER REFERENCES term (id),

term2_id INTEGER REFERENCES term (id),

label VARCHAR

);

// another edge table

Term2GeneProduct (

term_id INTEGER REFERENCES term (id),

gene_product_id INTEGER REFERENCES gene_product (id)

);

#### Mapping directives for GO:

CREATE DATA_SOURCE go

(

fullname ‘gene ontology’,

reference ‘http://www.geneontology.org’,

description ‘gene ontology is a controlled vocabulary …’

);

CREATE GRAPH go_graph

(

version STRING VALUE ‘go-11-2004’,

… other meta-data …

) SOURCE go;

IMPORT NODE FROM term

(

id AS ATTRIBUTE,

name AS ATTRIBUTE,

term_type AS ATTRIBUTE,

definition AS ATTRIBUTE

) GRAPH go_graph // indirectly, it suggests that this term is from source GO.

PRIMARY IDENTIFIER (id, name);

// primary identifier is used to compare two nodes which may be from different sources.

// In this example, if two nodes have the same id and name, then merge them.

// store species as a separate table.

// skip field ‘lineage’. Rename ‘genus’ to ‘genus_name’.

IMPORT TABLE go_species FROM species (id, genus AS genus_name, species);

// if we want to import table ‘species’ as it is, just say:

// IMPORT TABLE FORM species;

IMPORT NODE FROM gene_product

(

id AS ATTRIBUTE,

name AS ATTRIBUTE entity_name,

// rename the property name. It is especially useful during node merge.

// for example, genes from MIPS have a property called ‘orf’, and genes from

// SGD has a property called ‘orfname’. Both ‘orf’ and ‘orfname’ refer to the open

// reading frame name of a yeast gene. By renaming ‘orfname’ to ‘orf’, it indicates

// that the mapping tool should merge these two fields.

species_id AS ATTRIBUTE REFERENCES go_species (id)

// this reference association will be stored in system catalog.

) GRAPH go_graph

PRIMARY IDENITIFER (name);

// The edge still means traditional edge. Internally it will be split and stored as

// connectorNode and two links.

IMPORT EDGE FROM term2term

(

term1_id AS SOURCE REFERENCE,

term2_id AS TARGET REFERENCE,

label AS ATTRIBUTE relationship // edge attribute

) DIRECTION directed

GRAPH go_graph;

// Here, REFERENCE suggests to follow the foreign key specified in the relational

// schema to identify the node in term table.

// edge table

IMPORT EDGE FROM term2GeneProduct

(

term1_id AS SOURCE REFERENCE,

gene_product_id AS SOURCE REFERENCE

) DIRECTION directed

GRAPH go_graph;

// simple one-to-many property.

IMPORT NODE ATTRIBUTE FROM gene_product_synonyms

(

gene_product_id AS NODE REFERENCE, // node identifier

synonym AS ATTRIBUTE

) GRAPH go_graph;

// properties are already in name-value pair.

IMPORT NODE ATTRIBUTE FROM gene_product_properties

(

gene_product_id AS NODE REFERENCE,

property_name AS ATTRIBUTE KEY,

property_value AS ATTRIBUTE VALUE

) GRAPH go_graph;

// hypernode for complex type of data.

IMPORT HYPERNODE FROM gene_product_family

(

gene_product_family_name AS ATTRIBUTE,

gene_product_id AS MEMBER NODE REFERENCE,

putative AS MEMBER ATTRIBUTE

// internally, ‘putative’ becomes a property of ‘member-of’ edge.

) GRAPH go_graph

PRIMARY IDENTIFIER (gene_product_family_name);

## Quering Language

### Introduction

1. Language Definition.
2. Querying examples.
3. Querying biological networks.

Querying language functionality is implemented as Querying Window, from 'Tools' menu, where users can type and run their queries. Results of user's queries are immediately visualized in the main window. Pull-down menu of the query window describes six different types of queries as examples.

To run these queries, start from applying annotations to your graph. To check what kind of annotations are applied to your nodes/edges just right click on them. If 'Node Attribute Browser' does not appear, you need to download [yeast-context.jar](http://brak.sdsc.edu/pub/BiologicalNetworks/jnlp/cyto/yeast-context.jar) . Save it on your computer and upload through 'Plugins'->'Load plugins from Jar File' submenu. Note, for your plugins to work, they should be loaded before opening your graphs.

Apply your ontology annotations: GO, KEGG,.. . Click on the Ontology icon of the menu and add levels you are going to search by your queries. In general case of querying you need to apply all ontology levels.

After all annotations and ontologies are added you can start querying by clicking on 'Query Engine' from 'Tools' menu.

Be aware of next: **The Query must EXACTLY satisfy the language syntax.**

Be sure your terms are correctly spelled.

End your query with ';' and spell ontology terms dividing words by "_" like: "DNA_binding", "protein_serine/threonine_kinase", "general_transcriptional_repressor".

Note, queries searching through all GO tree (like descendants of highter ontology levels binding, enzyme, trancription_regulator) execute longer than simple queries (like children, parent, etc.).

### Language Definition

• //SELECTSELECTStatement

::= SELECT ("P_UNION" | "P_INTERSECTION" | "P_DIFFERENCE") SELECTSELECT

::= "SELECT" ("DISTINCT")? SELECTList FROMClause [WHEREClause]SELECTList

::= SELECTItem(","SELECTItem)*SELECTItem::=

(AggFunction"("ReturnType")")|(ReturnType)

•//FROMFROMClause

::= "FROM" ( Database(sb) "." DAG(sb) ( "AS" (f = )

(<K_COMMA>)? )? )*Database::= <S_IDENTIFIER>DAG::=<S_IDENTIFIER>

•//WHEREWHEREClause

::= "WHERE" SQLExpressionSQLExpression::=SQLPrimaryExpression(sb)

(","SQLPrimaryExpression(sb))*SQLPrimaryExpression::=

(( <S_IDENTIFIER> "." <S_IDENTIFIER> ) | <S_IDENTIFIER> ) "="

(( <S_IDENTIFIER> "." <S_IDENTIFIER> ) | NodeFunctionExp |

("REG_EXP" "(\"" LinkType(sb) ) ) )

### Querying examples

You can see the language syntax from the list of following examples:

1.a

SELECT path_bindings(a)

FROM dag(x,y,z)

WHERE a = idlist(1|2|3|999)

1.b

SELECT path_bindings(a//b)

FROM dag(x,y,z)

WHERE a = idlist(1|3),

b = idList(16|)

2.a

SELECT path_expression(a//b)

FROM dag(x,y,z)

WHERE a = idlist(1|3),

b = idList(16|)

2.b

SELECT full_path_expression(a//b)

FROM dag(x,y,z)

WHERE a = idlist(1|3),

b = idList(16|)

3.a

SELECT full_path_expression(a//b/c)

FROM dag(x,y,z)

WHERE a = nameEquals(nervous_system) union nameEquals(pns),

b = idlist(31|57|),

c=idlist(22|21)

3.b

SELECT path_bindings(a//b/c)

FROM dag(x,y,z)

WHERE a = nameEquals(nervous_system) union nameEquals(pns),

b = idlist(31|57|),

c = idlist(22|21)

4

SELECT full_path_expression(a//b/c//d)

FROM dag(x,y,z)

WHERE a = idlist(1|) difference idList(2|),

b = idlist(48|),

c = idlist(5|31|67|3),

d = idlist(12|)

5.a

SELECT path_bindings(a//b, c//d)

FROM dag(x,y,z)

WHERE a = nameEquals(nervous_system),

b = idList(19|20|),

c = idlist(46|),

d = nameEquals(dendrite)

5.b

SELECT path_expression(a//b), full_path_expression(c//d)

FROM dag(x,y,z)

WHERE a = nameEquals(nervous_system),

b = idList(19|20|),

c = idlist(46|),

d = nameEquals(dendrite)

5.c

SELECT path_bindings(a{l1}//b{l2}//c//d{l3}/e//f, g//h{l4}//i), path_expression(j{l5}//k/l)

FROM dag(database,username,passwd)

WHERE a = idlist(12|13|14) intersect nameEquals(protein) union idList(4|),

l3= "isa",

b = idList(23|24|25)

6.

SELECT full_path_expression(a{l1}//b)

FROM dag(x,y,z)

WHERE a = idlist(1|),

b = idlist(31|57|),

l1 = "isa"

7.

SELECT path_expression(a{l1}//b{l2}/c)

FROM dag(x,y,z)

WHERE a = idlist(1|),

b = idlist(49|50|48|52|),

c = idlist(5|31|67|3),

l2 = "has"

8.

SELECT full_path_expression(a{l1}//{l2}//{l3}b{l4}//d)

FROM dag(x,y,z)

WHERE a = idlist(23),

b = idList(49),

d = idlist(12),

l2 = "has",

l3 = "has",

l4 = "has"

### Querying biological networks

1. SELECT path(a)

FROM Yeast.go

WHERE a = descendant(nameEquals$DNA_binding);

Select a subnetwork of genes whose GO annotations fall under descendants of ‘DNA_binding’ term in GO hierarchy.

1. SELECT path(a)

FROM Yeast.go

WHERE a = children (nameEquals$protein_kinase);

Select a subnetwork of genes whose GO annotations fall under children of ‘protein_kinase’ term in GO hierarchy.

1. SELECT path(a)

FROM Yeast.go

WHERE a = parent (nameEquals$acid_phosphatase);

Select a subnetwork of genes whose GO annotations fall under parent of ‘acid_phosphatase’ term in GO hierarchy.

1. SELECT path_bindings(a//b)

FROM Yeast.go

WHERE a = children (nameEquals$protein_kinase);

b = parent (nameEquals$protein_threonine/tyrosine_kinase);

Select a binding (union) of two subnetworks one of genes whose GO annotations fall under children of ‘protein_kinase’ term in GO hierarchy and another whose GO annotations fall under parent of ‘protein_threonine/tyrosine_kinase’ term in GO hierarchy .

1. "Find colocalized proteins grouped by location".

SELECT p.location, set(p)

FROM yeastGraphDB G(N, E)

WHERE p:N and p.type = ’protein’

GROUP BY p.location;

Here, the from clause refers to a graph. Further, thanks to the grouping condition, the output is a nested relation instead of a graph, where due to the inner structuring element set, this query produces a set of tuples (genepairs) for every binding of location.

1. In the next query, we use graph operations in the body of the query, and the return data type is a graph. “Find networks of colocalized proteins that are parts of some protein complex and are connected by either a 2-hybrid (y2h) edge or a comimmunoprecipitation (coIP) edge.”

SELECT graph(N2(n.name, n.source), E2(e.label,e.source))

FROM yeastGraphDB G1(N, E)

WHERE n:N and c:N and e:E

and n.type << ’protein’

and c.type = ’protein complex’

and (e.label = ’y2h’ or e.label = ’coIP’)

and pathExpr(G1, c//[member of]n) = true

The query declares a variable c whose type is protein complex.

The query returns a graph, whose nodes n should be tuples with the attributes name and source (i.e., data source), and whose edges e should have a label and a source from which that edge was known. Recall that the system will convert this to a query on a connector node. The << operation specifies that the type of the node should be “under” protein in the node type hierarchy. The last line should be read as “n has an edge whose label has the value member, and this edge points to c”, where c is declared before. Note that we did not mention the relationship between nodes n and edges e, namely, an instance of the returned edge set e connects instances of the returned node set n. This constraint, expressed as n.edge = e, is implied by the construct of line 2, where n and e are constrained to be parts of the same graph.

1. Finally, we present an example from systems biological analysis that uses the graph-theoretic attributes. In this example, we take two subnetworks b1 and b2 produced by two subqueries, each using an aggregate graph function. For each network, the query computes the distribution of the betweenness centrality of the nodes of the respective graphs, and then uses the F-test to compare them.

WITH b1 AS (

SELECT distribution(betweenness centrality(*,0.05))

FROM yeastGraphDB G1(N, E)

WHERE n:N and e:E and

n.source IN (’Gavin-DB’, ’Ito-DB’, ’Tong-DB’)

and n.degree() > 2),

b2 AS (...)

SELECT F-test(b1, b2)

FROM b1, b2, stat-source;

Due to a heavy use of statistical operations, a number of statistical operations have been packaged in a source called stat-source. The function between-centrality produces a bag of values corresponding to the betweenness centrality of all nodes satisfying the remaining constraints. The function distribution takes a set of values and a bucket size and outputs a histogram, which is known to the system as a basic statistical data type defined as a table of 2-tuples {category, count} – here the category comes from the number of distinct values of the centrality measure with a bucket size of 0.05.

## Data Visualization

**PathSys** data visualization is done through the Client Side Application, [**BiologicalNetworks**](http://brak.sdsc.edu/pub/BiologicalNetworks/Software), which implements all business logic and a significant part of the user interface.
Sound *Data Model* together with strong *Ontology* capturing a big variety of biological data types is crucial for modeling capabilities of the system. But networks of molecular interactions derived from current biological data are incomplete and complicated. Complete network is clearly beyond human perception. Therefore different levels of *abstractions* are necessary to make effective analysis of cellular processes and dealing with complexity better.
One of such abstractions presented in **PathSys&BiologicalNetworks** is Meta-Node (or Graph Node).
Representing a cellular pathway as a single process or grouping related processes under a certain cellular mechanism enhance the comprehensibility of the networks of events. Grouping examples presented in **PathSys&BiologicalNetworks** are: domain structure of a protein, complex of multidomain proteins, chromosomal regions (promoters, binding sites, etc.) bound to a gene or group of genes, protein with its phosphorylation sites, gene transcription and mRNA translation or degradation mechanisms, whole metabolic pathway etc. Remember that grouping could be done over any data type object from *Node Types* hierarchy. Since the data on cellular processes is not complete, different levels of information may be available for certain events. In case where it is not identified which state among a set of states constitutes the *substrate*, *product* or *effector* of a process, or where target process of an effector is obscure, we may need to abstract these states or/and processes in a single state/process to represent the available information despite its incomplete nature (see Figure1 below).


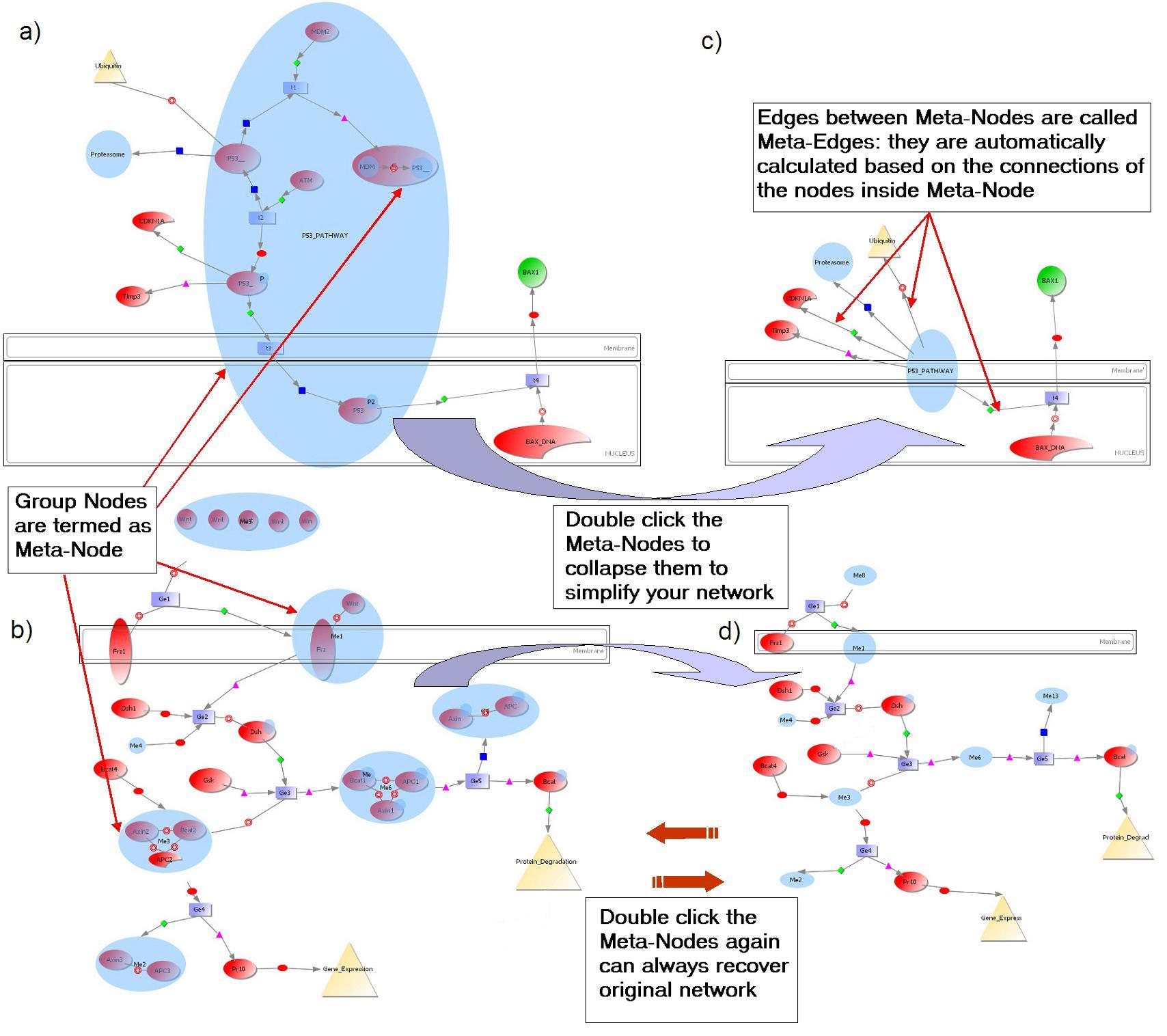


*Figure1. Abstraction helps better handling of complex information. For instance, part of a pathway may be collapsed to simplify relatively more complex graph (a->c, b->d). Two types of abstractions for representing information of incomplete nature: State and process abstraction. In addition (b, d) there are regular abstractions, “protein degradation”, “gene expression”, etc.*

## Case studies

1. Filtering procedure. Construction of high confidence integrated network
2. Retrieving of Cell Cycle Network.
3. Retrieving of Complex Interaction Network.
4. [Intermediate Results and Query execution Statistics.](http://brak.sdsc.edu/pub/BiologicalNetworks/PathSys/#t1_4)
5. Sample Queries and Results.
6. High-confidence MAPK network.

### Filtering procedure. Construction of high confidence integrated network.

To obtain high confidence integrated network we took next steps of filtering.

- Protein-protein interactions from MIPs were filtered to remove high-throughput (HTP) interactions contributed by yeast two-hybrid (y2h) and co-immunoprecipitation (co-IP) studies to construct MIPS HC (1207 nodes, 1785 edges).
- To get high confidence interactions (HTP HC all) from the high throughput protein-protein interactions, we took the union of two y2h data sets (Uetz et al. (2000) and Ito et al. (2001)) and its intersection with union of two co-IP data sets (Gavin et al. (2002) and Ho et al. (2002)), using matrix interpretation for co-IP data.
- High confidence DNA-protein network (MIT HC, 2420 nodes, 4365 interactions) was constructed from Lee et al. (2002) data filtered for a p-value threshold of 0.001.
- Genetic interactions from MIPs and Tong et al. (2001, 2004) were added to the high confidence DNA- protein data and all the interactions form this data set that were supported by at least one high throughput protein- protein interaction were used to construct genetic HC (289 nodes, 490 interactions).
- A high confidence, integrated interaction network (All HC) was derived by taking the union of MIPS HC, HTP HC all and genetic HC (1469 nodes, 2997 interactions, connected component of 1037 nodes).


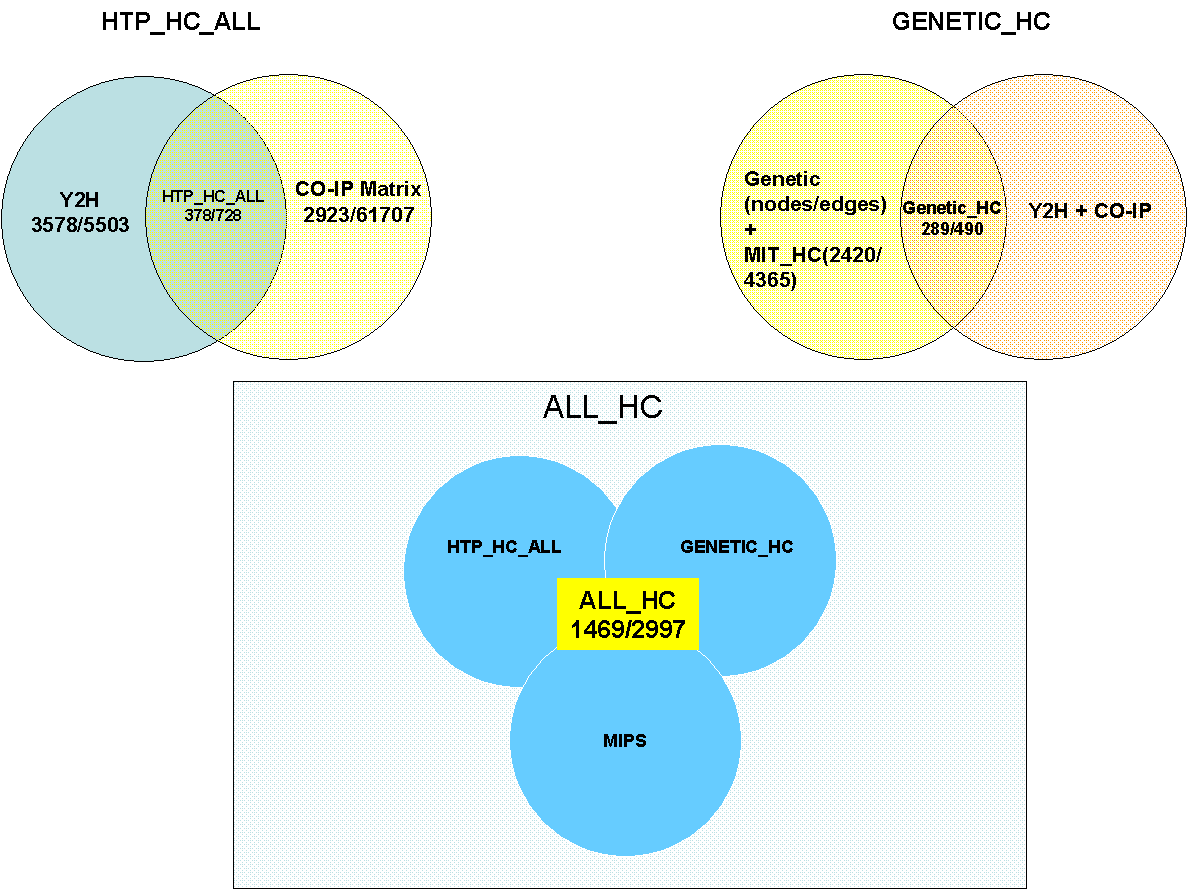


***Figure 1. Venn diagram summarizing data filtering procedures.***

- **HTP_HC_ALL:** high confidence physical interaction network supported by high throughput (HTP) experiment. The interaction must be supported by both Y2H and CO-IP/complex data. Different from HTP_HC, the network is not filtered with co-localization data. Graph file: [htp_hc_all.sif](http://pamina2.sdsc.edu/yeast/query_062004/htp_hc_all.sif)
- **all_hc** union of htp_hc_all, mips_hc and genetic_hc. Graph file: [all_hc.sif](http://pamina2.sdsc.edu/yeast/query_062004/all_hc.sif)
- **genetic_HC:** take HTP protein-protein interaction (Y2H, CO-IP or complex), pick those interactions that are supported by either MIPs genetic or HTP genetic data (Tong or MIT (cutoff < 0.001)). Graph file: [genetic_hc.sif](http://pamina2.sdsc.edu/yeast/query_062004/genetic_hc.sif)
- **MIPs_HC**: high confidence physical interaction network from MIPs. The interaction must be supported by biochemical data. If the interaction is supported only by high throughput experiment, it is not included. Graph file: [mips_hc.sif](http://pamina2.sdsc.edu/yeast/query_062004/mips_hc.sif)
- **ItoUetz_GENETIC_INTERSECT**: Intersection of (Ito Union Uetz) with (2 genetic Tong's data Union genetic_mips). Graph File: [itouetz_genetic_intersect.sif](http://pamina2.sdsc.edu/yeast/query_062004/itouetz_genetic_intersect.sif)
- **FYI:** Evidence for dynamically organized modularity in the yeast protein-protein interaction network. Vidal et al. 2004 Nature Vol430:88-93. supplement table 1. Graph File: [FYI.sif](http://pamina2.sdsc.edu/yeast/query_062004/FYI.sif)
- **HMI_network.sif:** union of MIPs_HC, htp_hc_all and itouetz_genetic_intersect. Graph File: [HMI_network.sif](http://pamina2.sdsc.edu/yeast/query_062004/HMI_network.sif)
- **FYI_HMI:** Intersection of FYI network and HMI_network. Interactions only form FYI and HMI are labeled as FYI and HMI respectively and the ones shared by both, represented as a single edge labeled as 'both'. Result: [FYI_HMI.sif](http://pamina2.sdsc.edu/yeast/query_062004/FYI_HMI.sif) .


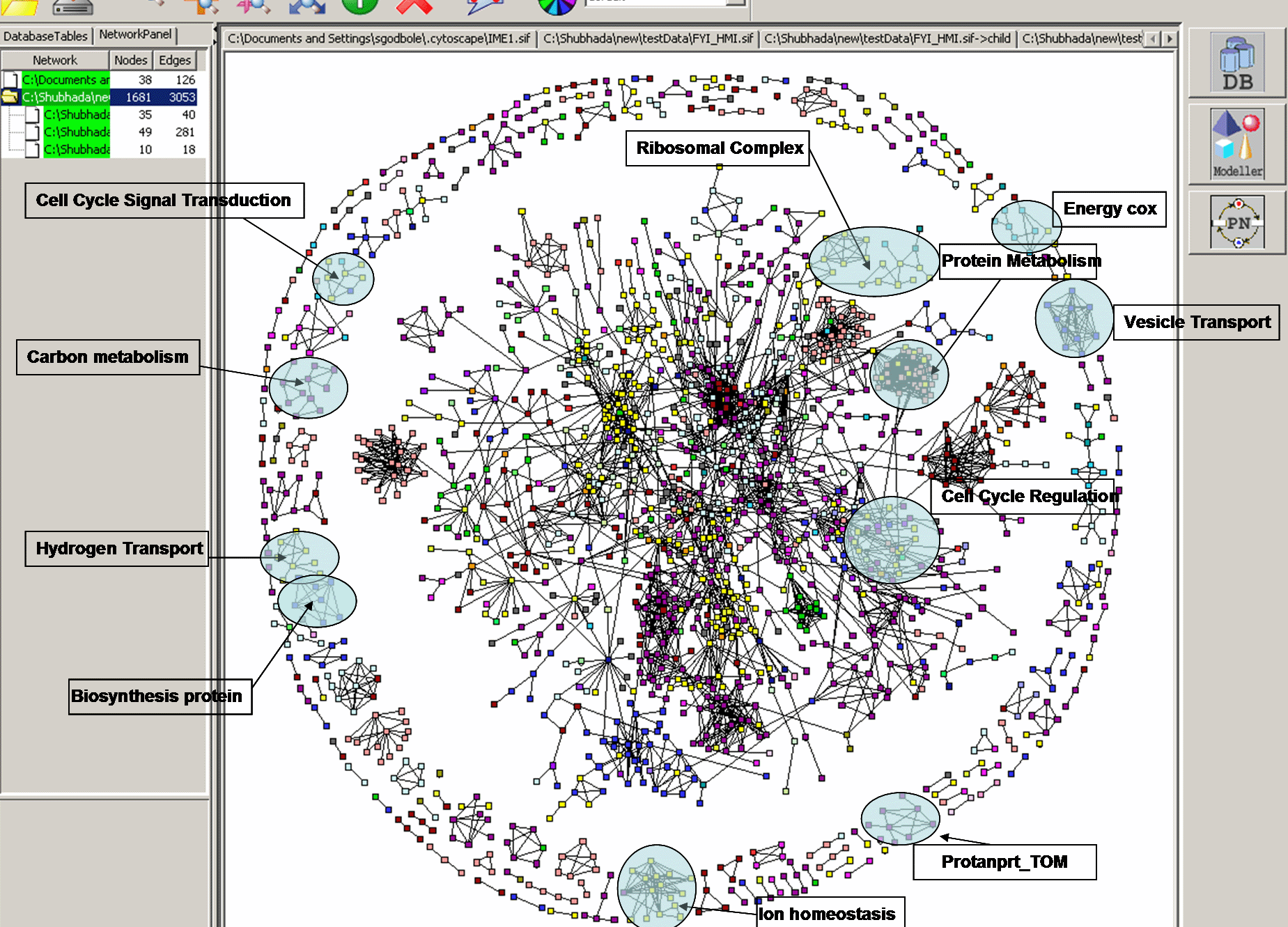


Figure 2a. Intersection of FYI network and HMI_network incorporating MIPS complexes and computational predictions (Han et al, 2004) colored due to GeneOntology annotation.

###
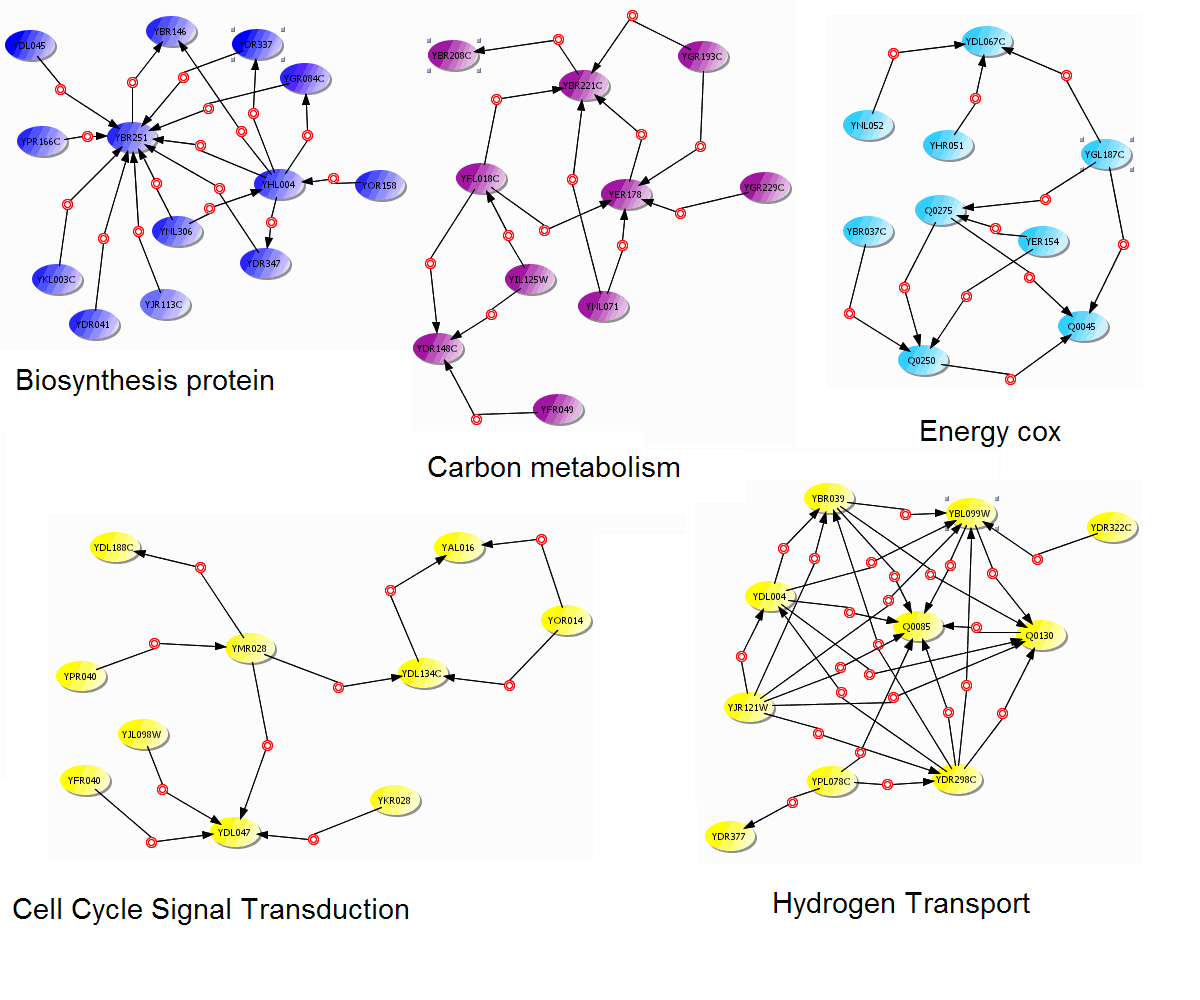

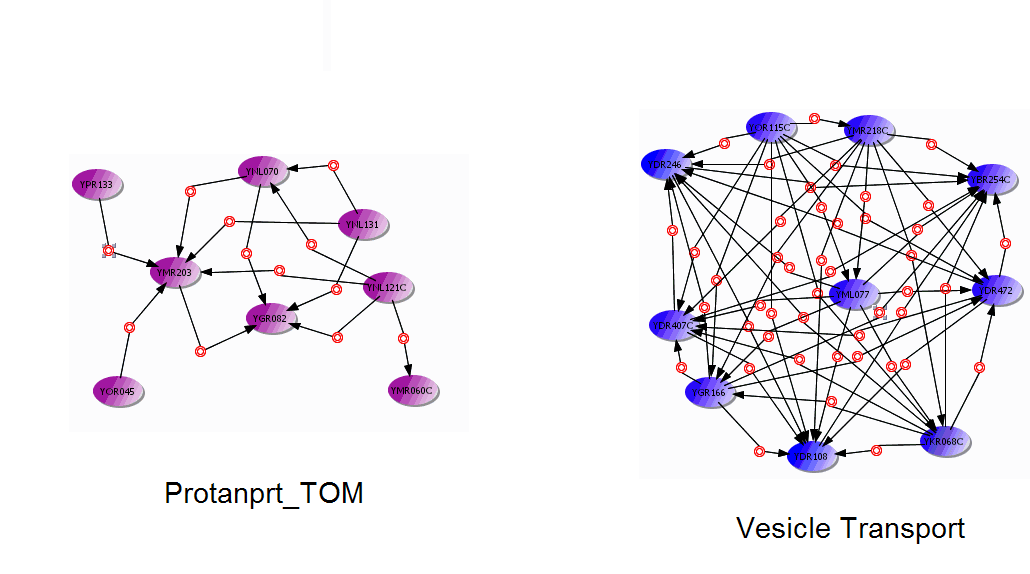


Figure 2b. Functionally related gene groups in FYI_HMI interaction network.

Figure 2c. Functionally related gene groups in FYI_HMI interaction network.

### Retrieving of Cell Cycle Network

This query demonstrates the use of PathSys as pre-screening tool for defining literature searches by quickly summarizing and reviewing the molecular interactions as well as transcriptional regulatory information for genes involved in a particular cellular process. Go biological process annotation was applied to a large network derived by union of all_hc and MIT_HC (MHM_HC) which was then filtered for genes involved in ‘cell cycle’. The resulting network shows well-known functional modules involved in the cell cycle such as DNA replication, DNA packaging, degradation of cyclins, chromosome segregation, DNA repair, etc. In addition it reveals cell cycle related transcription factors such as MCM1, ABF1, regulating their target genes. Even though the DNA protein interactions are derived from high-throughput datasets filtered to reduce false positives, caution should be used in interpreting the results, The related reference source for each interaction can then be obtained from the edge attribute information.

 **MHM_HC:** Union of MIT_HC, HTP_HC_ALL and MIPS_HC. (MHM stands for MIT_HTP_MIPS). Graph file: [mhm_hc.sif](http://pamina2.sdsc.edu/yeast/query_062004/mhm_hc.sif).

### Retrieving of Complex Interaction Network

| Complex _ID | BC value | Complex annotation |
| --- | --- | --- |
| 440.30.10 | 0.25024954 | mRNA splicing |
| 480.1 | 0.232740746 | SPB components |
| 270.20.10 | 0.162348718 | ctf19 protein complex |
| 510.40.20 | 0.158207356 | SRB mediator complex |
| 440.30.10.20 | 0.131013265 | prp 9/11/21complex |
| 270.20.40 | 0.12936989 | ndc80 protein complex |
| 260.50.10 | 0.0766123 | tSNARES |
| 140.20.20 | 0.066129342 | actin associated proteins |
| 480.2 | 0.061237633 | SPB associated proteins |
| 445.1 | 0.060252701 | SCF_CDC4 complex |
| 230.20.20 | 0.054251563 | SAGA |
| 510.190.10.20.10 | 0.054251563 | SAGA |
| 510.40.10 | 0.050920464 | RNA polII |
| 140.30.20 | 0.049608979 | tubulin associated proteins |
| 410.3 | 0.049004938 | pre-replication |

TABLE 1. Fifteen highest BC (betwenness centrality) complexes with BC values and their functional annotations

###
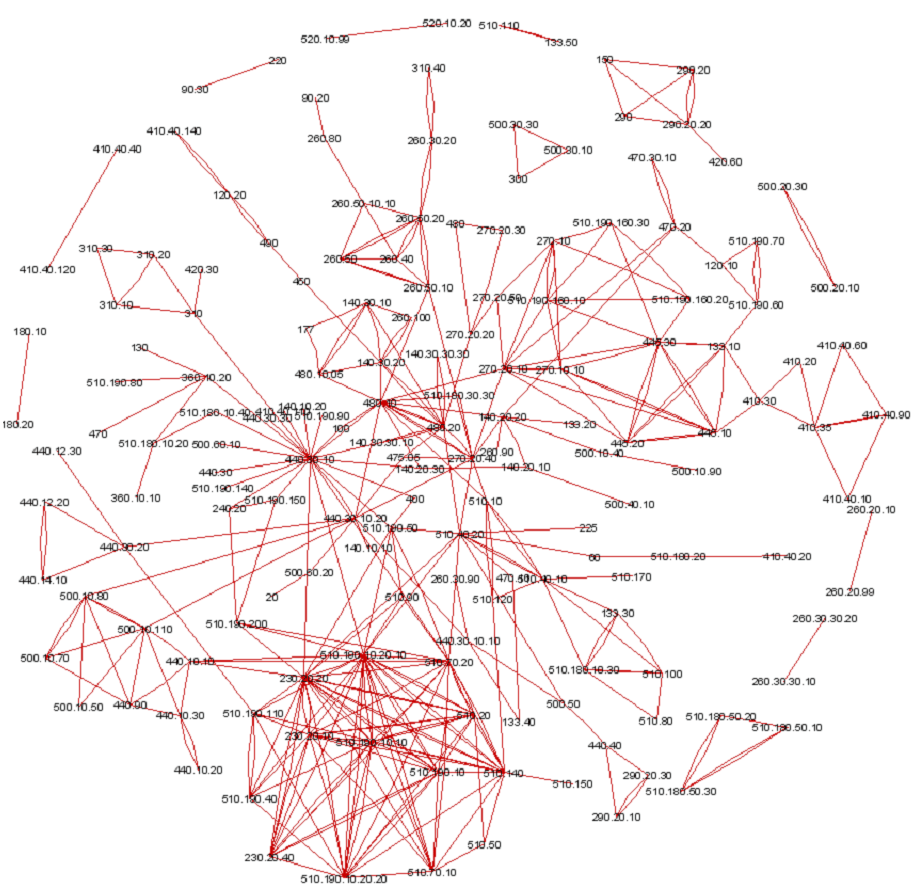


Figure 3. In this network with 164 nodes and 482 interactions, each node represents a protein complex identified by a complex_ID label from MIPS and edges are inter-complex protein-protein interactions from high-confidence HMI network.

###
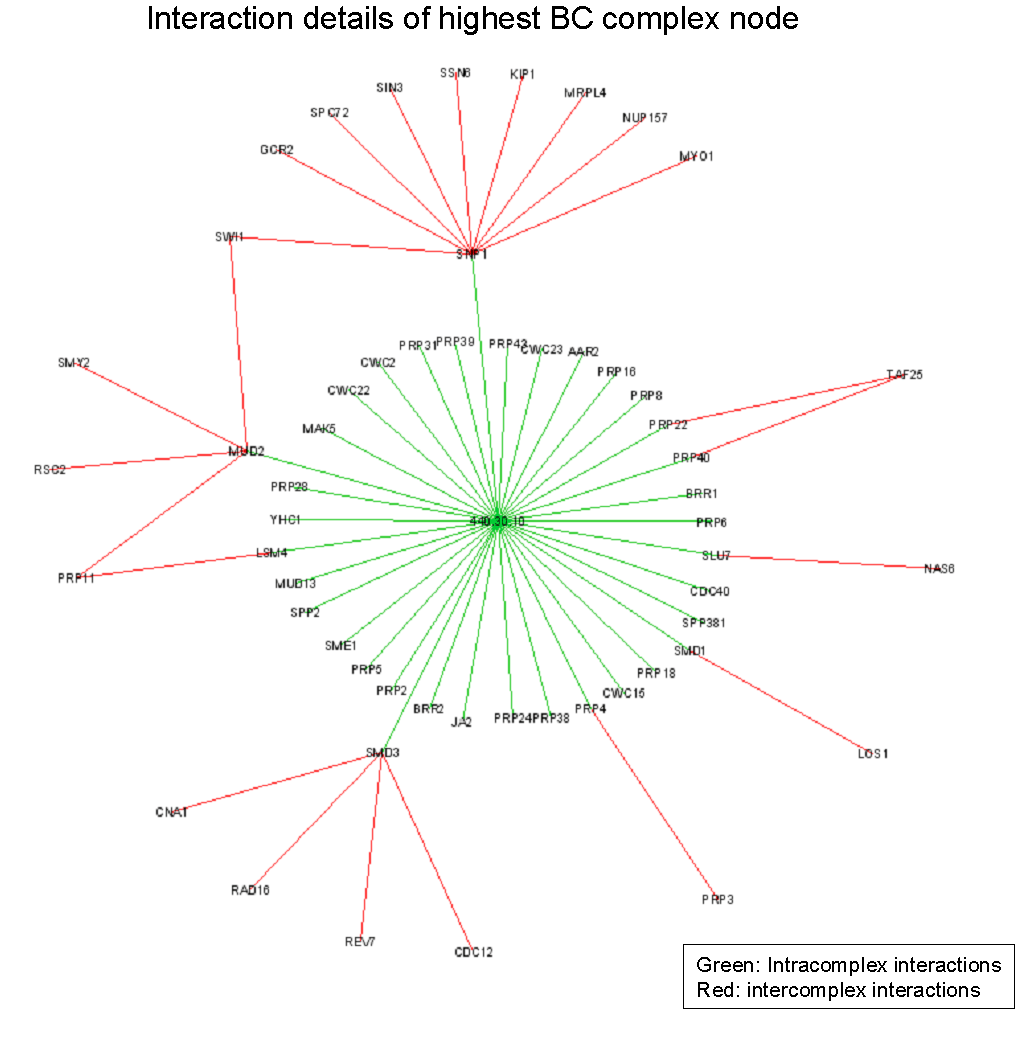


Figure 4. Interaction details on highest BC complex node, where in green are colored intracomplex interactions and in red- intercomplex interactions.

### Intermediate results and Query execution Statistics.

In the integrated graph, there are 25597 nodes and 1956530 edges.

- Number of gene pairs which are co-localized and physically interacted, verified by 2-hybrid: **1596**
- Number of complex/gene pairs which are co-localized and physically interacted, verified by co-ip/complex data: **790**
- Number of gene pairs which are co-localized and physically interacted, verified by 2-hybrid and co-ip/complex: **350**
- Number of gene pairs in MIT gene-regulator data (P_VALUE >=0): **708510**
- Number of gene pairs in MIT gene-regulator data (P_VALUE >= 0.8): **166434**
- Number of proteins at each cellular location:
- ER 295
- ER to Golgi 6
- Golgi 40
- NA 2062 -- excluded from all queries
- actin 32
- ambiguous 233
- bud 69
- bud neck 93
- cell periphery 158
- cytoplasm 1745
- early Golgi 52
- endosome 49
- late Golgi 46
- lipid particle 23
- microtubule 17
- mitochondrion 517
- nuclear periphery 52
- nucleolus 143
- nucleus 1336
- peroxisome 21
- punctate composite 139
- spindle pole 62
- vacuolar membrane 60
- vacuole 163

All intermediate networks are described below:

- **MIPs_HC:** high confidence physical interaction network from MIPs. The interaction must be supported by biochemical data. If the interaction is supported only by high throughput experiment, it is not included. Graph file: [mips_hc.sif](http://pamina2.sdsc.edu/yeast/query_062004/mips_hc.sif)
  **Fraction of edges returned by query: 0.09%**

**Execution Time: 12 sec.**

- **HTP_HC:** high confidence physical interaction network supported by high throughput (HTP) experiment. The interaction must be supported by both Y2H and CO-IP/complex data. The pair of proteins involved in the interaction must also be co-localized. The list of HTP data sets is shown [here](http://mips.gsf.de/proj/yeast/tables/interaction/). The protein localization data set is from UCSF. Graph file: [htp_hc.sif](http://pamina2.sdsc.edu/yeast/query_062004/htp_hc.sif).
  **Fraction of edges returned by query: 0.02%**

**Execution Time: 37 sec.**

#### BioNetSQL Query:

WITH htp_pi AS (

SELECT graph(e)

FROM yeastGraphDB G(N, E)

WHERE e:E and e.label = 'physical' and e.reference in ('htp_ref1', 'htp_ref2', ...) )

SELECT graph(e2)

FROM htp_pi G2(N2, E2)

WHERE e2:E2 and n2a:N2 and n2b:N2 and n2=e2.source and n3=e2.target and n2.location=n3.location;

- **HTP_HC_ALL:** high confidence physical interaction network supported by high throughput (HTP) experiment. The interaction must be supported by both Y2H and CO-IP/complex data. Different from HTP_HC, the network is not filtered with co-localization data. Graph file: [htp_hc_all.sif](http://pamina2.sdsc.edu/yeast/query_062004/htp_hc_all.sif)

  **Fraction of edges returned by query: 0.04%**

**Execution Time: 32 sec.**

- **HTP_HC_NL:** high confidence physical interaction network supported by high throughput (HTP) experiment. The interaction must be supported by both Y2H and CO-IP/complex data. For each interaction, one or both members' location is unknown in UCSF localization data. We would like to know how many interactions in HTP_HC_ALL fail to pass co-localization filter due to incomplete data. Graph file: [htp_hc_nl.sif](http://pamina2.sdsc.edu/yeast/query_062004/htp_hc_nl.sif)

**Fraction of edges returned by query: 0.01%**

**Execution Time: 21 sec.**

- **query1d_HC1:** union of MIPs_HC and HTP_HC. high confidence physical interaction network. Graph file: [query1d_hc1.sif](http://pamina2.sdsc.edu/yeast/query_062004/query1d_hc1.sif)

**Fraction of edges returned by query: 0.11%**

**Execution Time**: 9 sec.

- **query1d_hc_intersect:** intersection of htp_hc and mips_hc. Graph file: [query1d_hc_intersect.sif](http://pamina2.sdsc.edu/yeast/query_062004/query1d_hc_intersect.sif)
  **Fraction of edges returned by query: 0.005%**

**Execution Time: 9 sec.**

- **genetic_HC:** take HTP protein-protein interaction (Y2H, CO-IP or complex), pick those interactions that are supported by either MIPs genetic or HTP genetic data (Tong or MIT (cutoff < 0.001)). Graph file: [genetic_hc.sif](http://pamina2.sdsc.edu/yeast/query_062004/genetic_hc.sif)

**Fraction of edges returned by query: 0.03%**

**Execution Time: 18 sec.**

- **query1d_all:** union of genetic_hc, mips_hc and htp_hc. Graph file: [query1d_all.sif](http://pamina2.sdsc.edu/yeast/query_062004/query1d_all.sif)

**Fraction of edges returned by query: 0.14%**

**Execution Time: 5 sec.**

- **ppi_hc:** union of htp_hc_all and mips_hc. Graph file: [ppi_hc.sif](http://pamina2.sdsc.edu/yeast/query_062004/ppi_hc.sif)
- **Fraction of edges returned by query: 0.13%**

**Execution Time: 3 sec.**

- **all_hc** union of htp_hc_all, mips_hc and genetic_hc. Graph file: [all_hc.sif](http://pamina2.sdsc.edu/yeast/query_062004/all_hc.sif)
- **Fraction of edges returned by query: 0.15%**

**Execution Time: 7 sec.**

- **DP_genetic:** Get all interactions in MIT (p<0.001) or CSH (appropriate cut-off) which are supported by genetic interactions either from MIPs or Tong's. Graph file: [dp_genetic.sif](http://pamina2.sdsc.edu/yeast/query_062004/dp_genetic.sif).
  **Fraction of edges returned by query: 0.0005%**

**Execution Time: 18 sec.**

- **all_hcdp:** union of all_hc and DP_genetic. Graph file: [all_hcdp.sif](http://pamina2.sdsc.edu/yeast/query_062004/all_hcdp.sif).

**Fraction of edges returned by query: 0.15%**

**Execution Time: 4 sec.**

- **htp_ppi_2:** get all the protein-protein interactions from HTPs that are supported by either both Y2H datasets or both CO-IP datasets (using matrix model, complex data from MIPS) Graph file: [htp_ppi_2.sif](http://pamina2.sdsc.edu/yeast/query_062004/htp_ppi_2.sif).
- **Fraction of edges returned by query: 0.24%**

**Execution Time: 23 sec.**

- **query1_final** union of all_hcdp and htp_ppi_2. Graph file: [query1_final.sif](http://pamina2.sdsc.edu/yeast/query_062004/query1_final.sif).

**Fraction of edges returned by query: 0.39%**

**Execution Time: 4 sec.**

- **MIT_HC:** MIT interactions at a cut of P<0.001. Each row in the result file is an interaction. The format is: DNA dna_protein:MIT Factor. Graph file: [mit_hc.sif](http://pamina2.sdsc.edu/yeast/query_062004/mit_hc.sif).

**Fraction of edges returned by query: 0.22%**

**Execution Time: 6 sec.**

- **MHM_HC:** Union of MIT_HC, HTP_HC_ALL and MIPS_HC. (MHM stands for MIT_HTP_MIPS). Graph file: [mhm_hc.sif](http://pamina2.sdsc.edu/yeast/query_062004/mhm_hc.sif).

**Fraction of edges returned by query: 0.35%**

**Execution Time: 5 sec.**

- **MP2M_HC:** Union of MIT_HC, HTP_PPI_2 and MIPS_HC. (MP2M means MIT-HTP_PPI_2-MIPS). Graph file: [mp2m_hc.sif](http://pamina2.sdsc.edu/yeast/query_062004/mp2m_hc.sif).

**Fraction of edges returned by query: 0.56%**

**Execution Time: 5 sec.**

- **UETZ_Y2H:** Uetz's yeast two hybrid data set (PubMed ID = 10688190). Data file: [uetz_y2h.sif](http://pamina2.sdsc.edu/yeast/query_062004/uetz_y2h.sif)
  **Fraction of edges returned by query: 0.05%**

**Execution Time: 5 sec.**

- **ITO_Y2H:** Ito's yeast two hybrid data set (PubMed ID = 11283351). Data file: [ito_y2h.sif](http://pamina2.sdsc.edu/yeast/query_062004/ito_y2h.sif)
  **Fraction of edges returned by query: 0.23%**

**Execution Time: 5 sec.**

- **UETZ_ITO_INTERSECT:** The intersection (common edges) of Uetz's and Ito's data. Graph file: [uetz_ito_intersect.sif](http://pamina2.sdsc.edu/yeast/query_062004/uetz_ito_intersect.sif)

**Fraction of edges returned by query: 0.008%**

**Execution Time: 5 sec.**

- **UETZ_ITO_UNION:** The union of Uetz's and Ito's data. Graph file: [uetz_ito_union.sif](http://pamina2.sdsc.edu/yeast/query_062004/uetz_ito_union.sif)
  **Fraction of edges returned by query: 0.28%**

**Execution Time: 5 sec.**

- **Gavin's complex data:** PubMed ID = 11805826; [gavin_complex.txt: Original complex clusters](http://pamina2.sdsc.edu/yeast/query_062004/gavin_complex.txt); [gavin_matrix.sif: Matrix model](http://pamina2.sdsc.edu/yeast/query_062004/gavin_matrix.sif)

**Fraction of edges returned by query: 1.6%**

**Execution Time: 5 sec.**

- **Ho's complex data:** PubMed ID = 11805837; [ho_complex.txt: Original complex clusters](http://pamina2.sdsc.edu/yeast/query_062004/ho_complex.txt); [ho_matrix.sif: Matrix model](http://pamina2.sdsc.edu/yeast/query_062004/ho_matrix.sif)

**Execution Time: 5 sec.**

- **GAVIN_HO_INTERSECT:** The common edges of Gavin's and Ho's Co-IP matrix data. Graph File: [gavin_ho_intersect.sif](http://pamina2.sdsc.edu/yeast/query_062004/gavin_ho_intersect.sif)

**Fraction of edges returned by query: 0.11%**

**Execution Time: 5 sec.**

- **ItoUetz_GENETIC_INTERSECT:** Intersection of (Ito Union Uetz) with (2 genetic Tong's data Union genetic_mips). Graph File: [itouetz_genetic_intersect.sif](http://pamina2.sdsc.edu/yeast/query_062004/itouetz_genetic_intersect.sif)

**Fraction of edges returned by query: 0.002%**

**Execution Time: 5 sec.**

- **FYI:** Evidence for dynamically organized modularity in the yeast protein-protein interaction network. Vidal et al. 2004 Nature Vol430:88-93. supplement table 1. Graph File: [FYI.sif](http://pamina2.sdsc.edu/yeast/query_062004/FYI.sif)
  **Fraction of edges returned by query: 0.13%**

**Execution Time: 5 sec.**

- **genetic_lethal:** union Tong's genetic data and MIPs genetic interactions, then keep those interactions that are labeled with "synthetic lethal". Graph File: [genetic_lethal.sif](http://pamina2.sdsc.edu/yeast/query_062004/genetic_lethal.sif)
  **Fraction of edges returned by query: 0.13%**

**Execution Time: 18 sec.**

- **FYI_genetic_intersect:** First, union Tong's genetic data and MIPs genetic interactions, then keep those interactions that are labeled with "synthetic lethal", and intersect them with FYI data set. Graph File: [FYI_genetic_intersect.sif](http://pamina2.sdsc.edu/yeast/query_062004/FYI_genetic_intersect.sif)

**Fraction of edges returned by query: 0.004%**

**Execution Time: 5 sec.**

- **HMI_network.sif:** union of MIPs_HC, htp_hc_all and itouetz_genetic_intersect. Graph File: [HMI_network.sif](http://pamina2.sdsc.edu/yeast/query_062004/HMI_network.sif)
  **Fraction of edges returned by query: 0.03%**

**Execution Time: 4 sec.**

- **HMI_complex_network**: 1. Get MIPS complexes, which are considered as a gold standard and are high-confidence (represented as single nodes); 2. For each of the component proteins, expand the network by adding interaction from our high-confidence HMI network only. Only add the interactions that are not involving the proteins in the same complex (inter-complex interactions only and not intra-complex). Results:[A. this network excludes inter-complex edges that also appear as intra-complex edges in different complexes](http://pamina2.sdsc.edu/yeast/query_032005/HMI_complex_a.sif), [B. the network includes such edges](http://pamina2.sdsc.edu/yeast/query_032005/HMI_complex_b.sif), [MIPS_complex only](http://pamina2.sdsc.edu/yeast/query_032005/mips_complex.sif), [network A except mips_complex edges,](http://pamina2.sdsc.edu/yeast/query_032005/HMI_complex_a_interxn.sif) [network B except mips_complex edges.](http://pamina2.sdsc.edu/yeast/query_032005/HMI_complex_b_interxn.sif)

**Fraction of edges returned by query: 0.12%**

**Execution Time: 15 sec.**

- **Essential ORFs:** In SGD, select those ORFs that have "inviable" phenotype in the systematic deletion study. Result: [essential_orf.txt](http://pamina2.sdsc.edu/yeast/query_062004/essential_orf.txt)

**Fraction of edges returned by query: 0.24%**

**Execution Time: 4 sec.**

- **MIPs_biochem_genetic_lethal:** Synthetic lethal genetic interactions in MIPs supported by biochemical data. Graph File: [MIPs_biochem_genetic_lethal.sif](http://pamina2.sdsc.edu/yeast/query_062004/MIPs_biochem_genetic_lethal.sif)

**Fraction of edges returned by query: 0.03%**

**Execution Time: 16 sec.**

- **HTP_genetic_lethal:** Synthetic lethal genetic interactions in MIPs supported by HTP data. Graph File: [htp_genetic_lethal.sif](http://pamina2.sdsc.edu/yeast/query_062004/htp_genetic_lethal.sif)

**Fraction of edges returned by query: 0.12%**

**Execution Time: 5 sec.**

- **MIT_CSH_HC:** Find CSH interactions that have mapped edges where gene and factor both are known (not null). Union them with MIT_HC. Graph File: [MIT_CSH_HC.sif](http://pamina2.sdsc.edu/yeast/query_062004/mit_csh_hc.sif)
  **Fraction of edges returned by query: 0.24%**

**Execution Time: 21 sec.**

- **MIT_CSH_HC_INTERSECT:** Find CSH interactions that have mapped edges where gene and factor both are known (not null). Intersect them with MIT_HC. Graph File: [mit_csh_hc_intersect.sif](http://pamina2.sdsc.edu/yeast/query_062004/mit_csh_hc_intersect.sif)
  **Fraction of edges returned by query: 0.002%**

**Execution Time: 5 sec.**

- **ORF_NO_PI:** Take All ORFs from SGD. Find those ORFs that have no protein-protein interaction in MIPs, Gavin, Ho, Ito, Uetz's data sets, and no dna-protein interactions in MIT_HC (cutoff<0.001) and TRANSFAC. Result: [orf_no_pi.txt](http://pamina2.sdsc.edu/yeast/query_062004/orf_no_pi.txt)

**Fraction of edges returned by query: 0.02%**

**Execution Time: 5 sec.**

- **NO_PI_GENETIC:** For each ORF in ORFs_NO_PI, find the genes genetically interacted with the ORF from TONG + MIPs_genetic. Result: [no_pi_genetic.sif](http://pamina2.sdsc.edu/yeast/query_062004/no_pi_genetic.sif)
  **Fraction of edges returned by query: 0.01%**

**Execution Time: 5 sec.**

- **NO_PI_PREBIND:** Find the interactions in PreBIND involving the ORFs in ORFs_NO_PI. Result: [no_pi_prebind.sif](http://pamina2.sdsc.edu/yeast/query_062004/no_pi_prebind.sif)

**Fraction of edges returned by query: 0.005%**

**Execution Time: 5 sec.**

- **DEGREE_FYI_SL:** neighbors of [degree outliers](http://pamina2.sdsc.edu/yeast/query_062004/outliers_1.txt) in FYI_union_SL network (SL: genetically lethal interactions). Result:[degree_fyi_sl.sif](http://pamina2.sdsc.edu/yeast/query_062004/degree_fyi_sl.sif)

**Fraction of edges returned by query: 0.005%**

**Execution Time: 5 sec.**

- **CC_FYI_SL:** neighbors of [clustering coefficient outliers](http://pamina2.sdsc.edu/yeast/query_062004/outliers_2.txt) in FYI_union_SL network. Result: [cc_fyi_sl.sif](http://pamina2.sdsc.edu/yeast/query_062004/cc_fyi_sl.sif)
  **Fraction of edges returned by query: 0.003%**

**Execution Time: 5 sec.**

- **BC_FYI_SL:** neighbors of [betweenness centrality outliers](http://pamina2.sdsc.edu/yeast/query_062004/outliers_3.txt) in FYI_union_SL network. Result: [bc_fyi_sl.sif](http://pamina2.sdsc.edu/yeast/query_062004/bc_fyi_sl.sif)

**Fraction of edges returned by query: 0.003%**

**Execution Time: 5 sec.**

- **DEGREE_FYI_DP:** neighbors of [degree outliers](http://pamina2.sdsc.edu/yeast/query_062004/outliers_4.txt) in FYI_union_DP network (DP=MIT_HC (MIT with cutoff < 0.001). Result: [degree_fyi_dp.sif](http://pamina2.sdsc.edu/yeast/query_062004/degree_fyi_dp.sif)

**Fraction of edges returned by query: 0.04%**

**Execution Time: 5 sec.**

- **CC_FYI_DP:** neighbors of [clustering coefficient outliers](http://pamina2.sdsc.edu/yeast/query_062004/outliers_5.txt) in FYI_union_DP network. Result: [cc_fyi_dp.sif](http://pamina2.sdsc.edu/yeast/query_062004/cc_fyi_dp.sif)
  **Fraction of edges returned by query: 0.001%**

**Execution Time: 5 sec.**

- **DEGREE_SL_DP:** neighbors of [degree outliers](http://pamina2.sdsc.edu/yeast/query_062004/outliers_6.txt) in SL_union_DP network. Result: [degree_sl_dp.sif](http://pamina2.sdsc.edu/yeast/query_062004/degree_sl_dp.sif)
  **Fraction of edges returned by query: 0.003%**

**Execution Time: 5 sec.**

- **OUTLIERS_NN_FDS:** neighbors of [the outliers](http://pamina2.sdsc.edu/yeast/query_062004/outliers.txt) in FYI_union_DP_union_SL network. Result: [outliers_nn_fds.sif](http://pamina2.sdsc.edu/yeast/query_062004/outliers_nn_fds.sif)

**Fraction of edges returned by query: 0.05%**

**Execution Time: 5 sec.**

- **OUTLIERS_FDS_NETWORK:** interactions between [the outliers](http://pamina2.sdsc.edu/yeast/query_062004/outliers.txt) in FYI_union_DP_union_SL network. Result: [outliers_fds_network.sif](http://pamina2.sdsc.edu/yeast/query_062004/outliers_fds_network.sif)

**Fraction of edges returned by query: 0.001%**

**Execution Time: 5 sec.**

- **FYI_HMI:** Intersection of FYI network and HMI_network. Interactions only form FYI and HMI are labeled as FYI and HMI respectively and the ones shared by both, represented as a single edge labeled as 'both'. Result: [FYI_HMI.sif](http://pamina2.sdsc.edu/yeast/query_062004/FYI_HMI.sif)

**Fraction of edges returned by query: 0.16%**

**Execution Time: 23 sec.**

- **MIN_NETWORK:** Given a list of [meiosis-related genes](http://pamina2.sdsc.edu/yeast/query_122004/min_net_nodes.txt), find the shortest paths between each pair (n*(n-1)/2 pairs). Union all shortest paths into one graph. Note: use cutoff<0.001 for MIT data. Result: [min_network.sif](http://pamina2.sdsc.edu/yeast/query_122004/min_network.sif) If we use cutoff < 0.005, the result min_network_005.sif is [here](http://pamina2.sdsc.edu/yeast/query_122004/min_network_005.sif).

**Fraction of edges returned by query: 0.005%**

**Execution Time: very long (~10 minutes)**

- **MIN_NETWORK_ALL:** In min_network, extract all [transcription factors](http://pamina2.sdsc.edu/yeast/query_122004/tf_nodes.txt) (those nodes which are descendents of "transcription regulator" (GO:0030528). Find each transcription factor's direct neighbors in CSH, MIT (cutoff<0.001) and TRANSFAC. Union the neighbors with min_network. Result: [min_network_all.sif](http://pamina2.sdsc.edu/yeast/query_122004/min_network_all.sif) Similarly, find neighborhood of [transcription factors](http://pamina2.sdsc.edu/yeast/query_122004/tf_nodes_005.txt) from min_network_005, and union them together to form [min_network_all_005.sif](http://pamina2.sdsc.edu/yeast/query_122004/min_network_all_005.sif)
  **Fraction of edges returned by query: 0.08%**

**Execution Time: 7 sec.**

- **GO Distance Matrix for yeast genes in FYI** [yeast_gene_list.txt](http://pamina2.sdsc.edu/yeast/query_032005/yeast_gene_list.txt) contains all genes in FYI network. For each pair of genes in the list, find their GO distance in Biological_Process, Cellular_Component, Molecular_Function subgraphs. If node C is the least common ancestor (LCA) of gene A and B, then the GO distance between A and B is Distance (A, C) + Distance(B, C). For example, if A and B has a common parent, then GO distance between A and B is 1+1 = 2. If A and B do not have common ancestor, their distance is -1. Results: GO distance matrix in [Biological Process](http://pamina2.sdsc.edu/yeast/query_032005/bp_matrix.txt), [Molecular Function](http://pamina2.sdsc.edu/yeast/query_032005/mf_matrix.txt), [Cellular Component](http://pamina2.sdsc.edu/yeast/query_032005/cc_matrix.txt).
  **Fraction of edges returned by query: not available**

**Execution Time: 42 sec.**

- **GO Distance Matrix for fly genes in BIND-FLY interaction network**. Similar to how we compute the yeast GO distance matrix, [fly_gene_name_list.txt](http://pamina2.sdsc.edu/yeast/query_032005/fly_gene_name_list.txt) contains all genes in BIND-FLY interaction network. Results: GO distance matrix in [Biological Process](http://pamina2.sdsc.edu/yeast/query_032005/fly_bp_matrix.txt), [Molecular Function](http://pamina2.sdsc.edu/yeast/query_032005/fly_mf_matrix.txt), and [Cellular Component](http://pamina2.sdsc.edu/yeast/query_032005/fly_cc_matrix.txt).

**Fraction of edges returned by query: not available**

**Execution Time: 37 sec.**

- **Interactions among peroxisome-related genes**: 1. [peroxisome-related genes](http://pamina2.sdsc.edu/yeast/query_032005/peroxisome_genes.txt); 2. [interactions among the genes](http://pamina2.sdsc.edu/yeast/query_032005/peroxisome_direct_interxn.sif) ([screenshot in Cytoscape](http://pamina2.sdsc.edu/yeast/query_032005/peroxisome_direct_interxn.gif)); 3. [union of shortest paths](http://pamina2.sdsc.edu/yeast/query_032005/peroxisome_unionSP.sif) between all pairs of peroxisome-related genes ([screenshot in BiologicalNetworks](http://pamina2.sdsc.edu/yeast/query_032005/peroxisome_unionSP.jpg));

**Fraction of edges returned by query: 0.07%**

**Execution Time: very, very long (about 1 day)**

#### BioNetSQL Query:

WITH peroxisome_genes AS (

SELECT n1

FROM yeastGraphDB G(N1, E1)

WHERE n1:N1 and n1:description like '%peroxisom%' )

SELECT union_of_shortest_paths(G2, peroxisome_genes)

FROM yeastGraphDB G2, peroxisome_genes;

### Sample Queries and Results

#### Query 1d:

Find physically interacted proteins. The interaction is verified by yeast two-hybrid. The protein pairs are either co-immunoprecipitated or co-existing in some complex. They are also co-localized. ([Results](http://pamina2.sdsc.edu/yeast/QUERY1D.HTML))

#### Query 1e:

Find proteins that are co-localized, but not appear in any complex, 2-hydrid or co-ip data. ([Results](http://pamina2.sdsc.edu/yeast/query1e.txt))

#### Query 2c:

Find gene pairs satisfying the following conditions:

(1) co-localized and physically interacted, verified by 2-hybrid and co-ip/complex data ([See Query 1D Results](http://pamina2.sdsc.edu/yeast/QUERY1D.HTML)).

(2) genetically interacted or one gene is regulated by the protein of the other gene (DNA-protein interaction) ([Results](http://pamina2.sdsc.edu/yeast/QUERY2C.HTML))

**NOTE:** If we use P_VALUE 0.8 as cutoff when we choose gene-regulator data from MIT database, the results are [here](http://pamina2.sdsc.edu/yeast/QUERY2C_SUB.HTML)

#### Query 2c-a:

Do the same query as Query 2c except that the condition1 is changed to: co-localized and physically interacted, verified by 2-hybrid data. ([Results](http://pamina2.sdsc.edu/yeast/QUERY2CA.HTML))

#### Query 2c-b:

Do the same query as Query 2c except that the condition1 is changed to: co-localized and physically interacted, verified by co-ip/complex data. ([Results](http://pamina2.sdsc.edu/yeast/QUERY2CB.HTML))

#### Query 2d:

- - result 2ab: gene pairs that are genetically interacted or one gene is regulated by the protein of the other gene (DNA-protein interaction, P_VALUE >= 0.8)
  - result 2d: result 1e (see query 1e) intersect result 2ab.
    ([Results](http://pamina2.sdsc.edu/yeast/QUERY2D_SUB.HTML))

**NOTE:** The graph may be too big to display, a text version is [here](http://pamina2.sdsc.edu/yeast/query2d_sub1.txt)

Download [query2d_new.sif](http://pamina2.sdsc.edu/yeast/query2d_new.sif) to display results in Cytoscape. The format is:

FACTOR pd DNA

**All query results are exported into an MS Excel file (**[**query_results.xls**](http://pamina2.sdsc.edu/yeast/query_results.xls)**). GO function and process annotation for each node is included.**

#### Find neighbors of Query 1d results:

For each subgraph that has more than 4 nodes, find the proteins/genes interacted with these nodes (MIT source is excluded).

| **subgraph node list** | **expanded graph (.sif)** |
| --- | --- |
|  |  |
| [sub1.txt](http://pamina2.sdsc.edu/yeast/query1d/sub1.txt) | [query1d_sub1.sif](http://pamina2.sdsc.edu/yeast/query1d/query1d_sub1.sif) |
| [sub2.txt](http://pamina2.sdsc.edu/yeast/query1d/sub2.txt) | [query1d_sub2.sif](http://pamina2.sdsc.edu/yeast/query1d/query1d_sub2.sif) |
| [sub3.txt](http://pamina2.sdsc.edu/yeast/query1d/sub3.txt) | [query1d_sub3.sif](http://pamina2.sdsc.edu/yeast/query1d/query1d_sub3.sif) |
| [sub4.txt](http://pamina2.sdsc.edu/yeast/query1d/sub4.txt) | [query1d_sub4.sif](http://pamina2.sdsc.edu/yeast/query1d/query1d_sub4.sif) |
| [sub5.txt](http://pamina2.sdsc.edu/yeast/query1d/sub5.txt) | [query1d_sub5.sif](http://pamina2.sdsc.edu/yeast/query1d/query1d_sub5.sif) |
| [sub6.txt](http://pamina2.sdsc.edu/yeast/query1d/sub6.txt) | [query1d_sub6.sif](http://pamina2.sdsc.edu/yeast/query1d/query1d_sub6.sif) |
| [sub7.txt](http://pamina2.sdsc.edu/yeast/query1d/sub7.txt) | [query1d_su b7.sif](http://pamina2.sdsc.edu/yeast/query1d/query1d_sub7.sif) |
| [sub8.txt](http://pamina2.sdsc.edu/yeast/query1d/sub8.txt) | [query1d_sub8.sif](http://pamina2.sdsc.edu/yeast/query1d/query1d_sub8.sif) |
| [sub9.txt](http://pamina2.sdsc.edu/yeast/query1d/sub9.txt) | [query1d_sub9.sif](http://pamina2.sdsc.edu/yeast/query1d/query1d_sub9.sif) |
| [sub10.txt](http://pamina2.sdsc.edu/yeast/query1d/sub10.txt) | [query1d_sub10.sif](http://pamina2.sdsc.edu/yeast/query1d/query1d_sub10.sif) |
| [sub11.txt](http://pamina2.sdsc.edu/yeast/query1d/sub11.txt) | [query1d_sub11.sif](http://pamina2.sdsc.edu/yeast/query1d/query1d_sub11.sif) |
| [sub12.txt](http://pamina2.sdsc.edu/yeast/query1d/sub12.txt) | [query1d_sub12.sif](http://pamina2.sdsc.edu/yeast/query1d/query1d_sub12.sif) |
| [sub13.txt](http://pamina2.sdsc.edu/yeast/query1d/sub13.txt) | [query1d_sub13.sif](http://pamina2.sdsc.edu/yeast/query1d/query1d_sub13.sif) |
| [sub14.txt](http://pamina2.sdsc.edu/yeast/query1d/sub14.txt) | [query1d_sub14.sif](http://pamina2.sdsc.edu/yeast/query1d/query1d_sub14.sif) |

#### Query 3:

For genes in cluster 4 of Esposito's sporulation microarray data, show those genes interact with each other. The interaction type could be protein-protein, DNA-protein or genetic interaction. For DNA-protein interaction, use P_VALUE > 0.8 as cutoff. (Result file: [query3.sif](http://pamina2.sdsc.edu/yeast/query3.sif))

**NOTE:** For DNA-protein edges, the first ORF is DNA and the second is the factor. For example:

YDR374C dna_protein YOL067C

YDR374C is the DNA bound by factor YOL067C.

#### Query 3a:

For genes in cluster 4 of Esposito's sporulation microarray data, show those genes interact with each other, AND co-localized. The interaction type could be protein-protein, DNA-protein or genetic interaction. For DNA-protein interaction, use P_VALUE > 0.8 as cutoff. (Result file: [query3a.sif](http://pamina2.sdsc.edu/yeast/query3a.sif))

#### Query 3b:

For genes in cluster 4 of Esposito's sporulation microarray data, show those genes interact with each other, but NOT co-localized. The interaction type could be protein-protein, DNA-protein or genetic interaction. For DNA-protein interaction, use P_VALUE > 0.8 as cutoff. (Result file: [query3b.sif](http://pamina2.sdsc.edu/yeast/query3b.sif))

#### Query 3c:

For genes in cluster 1,2,3 and 4 of Esposito's sporulation microarray data, show those genes interact with each other. The interaction type could be protein-protein, DNA-protein or genetic interaction. For DNA-protein interaction, use P_VALUE > 0.8 as cutoff. (Result file: [query3c.sif](http://pamina2.sdsc.edu/yeast/query3c.sif))

#### Query 3d:

For genes in cluster 1,2,3 and 4 of Esposito's sporulation microarray data, show those genes interact with each other, AND co-localized. The interaction type could be protein-protein, DNA-protein or genetic interaction. For DNA-protein interaction, use P_VALUE > 0.8 as cutoff. (Result file: [query 3d.sif](http://pamina2.sdsc.edu/yeast/query3d.sif))

#### Query 3e:

For genes in cluster 1,2,3 and 4 of Esposito's sporulation microarray data, show those genes interact with each other, but NOT co-localized. The interaction type could be protein-protein, DNA-protein or genetic interaction. For DNA-protein interaction, use P_VALUE > 0.8 as cutoff. (Result file: [query 3e.sif](http://pamina2.sdsc.edu/yeast/query3e.sif))

#### Query 4a:

Find all neighbors of SPO11. The interaction type could be protein-protein, DNA-protein or genetic interaction. For all these neighbors, find protein-protein and genetic interactions. (P_VALUE > 0.8. Restul file: [query 4a.sif](http://pamina2.sdsc.edu/yeast/query4a.sif))

#### Query 4b:

Find 2-nearest neighbors of SPO11. The interaction type is either protein-protein or genetic interaction. [query 4b.sif](http://pamina2.sdsc.edu/yeast/query4b.sif))

#### Query 5a:

Select those genes that show anything but "normal" in Enyenihi and Saunder's [sporulation defects data](http://pamina2.sdsc.edu/yeast/http://www.genetics.org/content/vol163/issue1/images/data/47/DC1/Table_1_of_total_results.xls), find any networks within them, with any possible interactions.
* [query5a_1.sif](http://pamina2.sdsc.edu/yeast/query5a_1.sif): Use P_VALUE > 0.8 as cutoff for MIT's DNA-protein interactions.
* [query5a_2.sif](http://pamina2.sdsc.edu/yeast/query5a_2.sif): Use P_VALUE > 0.95 as cutoff for MIT's DNA-protein interactions.

#### Query 5b:

In Esposito's cluster 1,2,3 and 4, there are total 202 genes. In Enyenihi's data, there are 479 genes displaying anything but 'normal' sporulation defects. Among these two groups of genes,

- - [23 genes](http://pamina2.sdsc.edu/yeast/query5b_1.txt) appear in both groups.
  - [179 genes](http://pamina2.sdsc.edu/yeast/query5b_2.txt) appear in only Esposito's cluster 1-4.
  - [456 genes](http://pamina2.sdsc.edu/yeast/query5b_3.txt) appear in only Enyenihi's subset.

Union these two groups of genes, and find any networks within them, with any possible interactions.

- - [query5b_4.sif](http://pamina2.sdsc.edu/yeast/query5b_4.sif): Use P_VALUE > 0.8 as cutoff for MIT's DNA-protein interactions.
  - [query5b_5.sif](http://pamina2.sdsc.edu/yeast/query5b_5.sif): Use P_VALUE > 0.95 as cutoff for MIT's DNA-protein interactions

**NOTE :** The network uses ONLY those genes in the group. If there is a path through some gene that is not in the group, the path is not picked up.

#### Query 6:

From Enyenihi's paper take uncharacterized ORFs that showed effect on IME1 induction ( listed in Table1.doc, select for IME1 induction less than ++++), find neighborhood of these genes. The goal is to see if they start relating to any nutritional/environmental signal transduction pathways. Hopefully, we can find between these genes and IME1.

**NOTE:** P_VALUE > 0.95 is used as cutoff for MIT's DNA-protein interactions.
* take those genes with effect less than ++++, find their nearest neighbors. ( [query6a.sif](http://pamina2.sdsc.edu/yeast/query6a.sif))
* take those genes with effect less than +++, find their nearest neighbors. ( [query6b.sif](http://pamina2.sdsc.edu/yeast/query6b.sif))
* take those genes with effect less than ++, in other words, 0 or +, find their nearest neighbors. ( [query6c.sif](http://pamina2.sdsc.edu/yeast/query6c.sif))

#### Query 7:

7-1: protein-protein interaction graph (excluding BIND and PREBIND) of those proteins whose genes are expressed in sporulation/meiosis during the first 6-8 hours. ([Results 7-1](http://pamina2.sdsc.edu/yeast/query7_1.sif))

7-2: The whole genome transcription factor-DNA interaction graph from MIT data, using P_VALUE cutoff 0.999 ([Results 7-2](http://pamina2.sdsc.edu/yeast/query7_2.sif))

7-3a: The union of query 7-1 and 7-2 ([Results 7-3a](http://pamina2.sdsc.edu/yeast/query7_3a.sif))

7-3b: The intersection of query 7-1 and 7-2: empty results

7-4: The genetic interaction graph, not including Tong's data. ([Results 7-4](http://pamina2.sdsc.edu/yeast/query7_4.sif))

7-5a: The union of query 7-1 and 7-4 ([Results 7-5a](http://pamina2.sdsc.edu/yeast/query7_5a.sif))

7-5b: The intersection of query 7-1 and 7-4 ([Results 7-5b](http://pamina2.sdsc.edu/yeast/query7_5b.sif))

7-6a: The union of query 7-2 and 7-4 ([Results 7-6a](http://pamina2.sdsc.edu/yeast/query7_6a.sif))

7-6b: The intersection of query 7-2 and 7-4 : empty results

7-7a: The union of query 7-1, 7-2 and 7-4 ([Results 7-7a](http://pamina2.sdsc.edu/yeast/query7_7a.sif))

7-7b: The intersection of query 7-1, 7-2 and 7-4 : empty results

#### Query IME:

List genes bound by IME4, order by P_VALUE. ([Results](http://pamina2.sdsc.edu/yeast/IME4_DB.txt))

#### Some data sets are [here](http://pamina2.sdsc.edu/yeast/data.html)

### High-confidence MAPK network


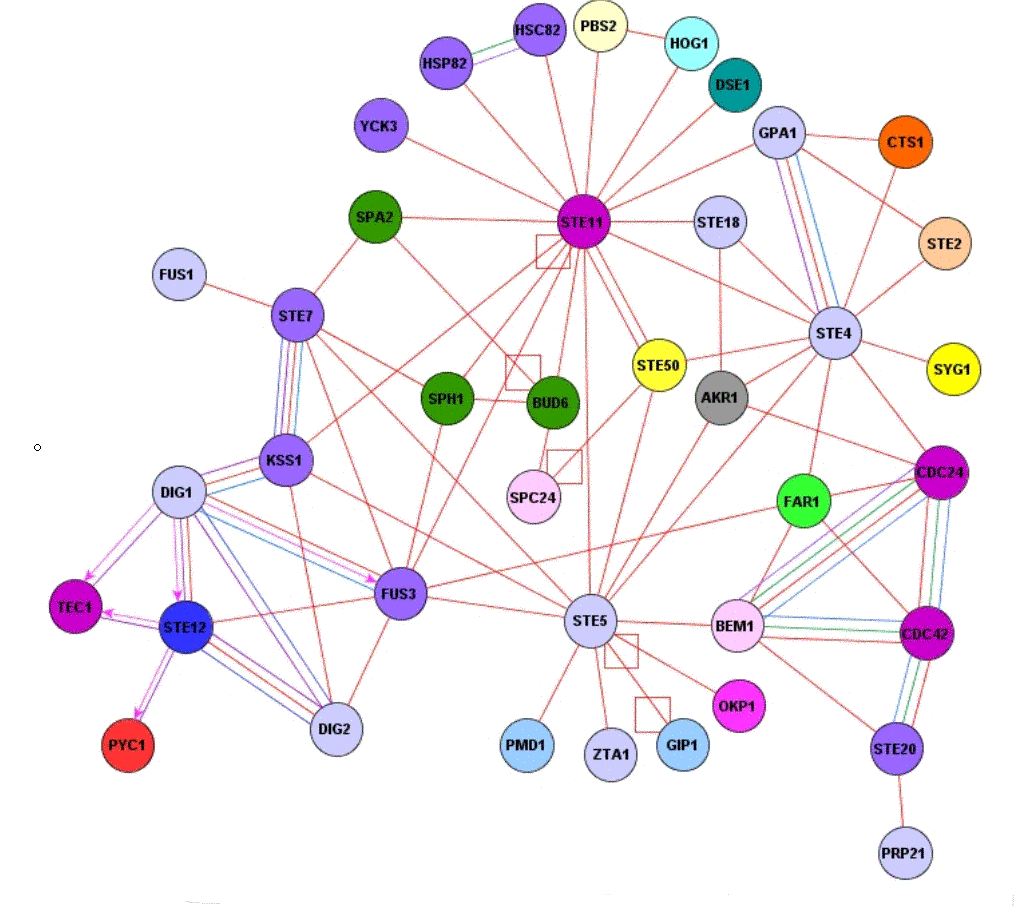
The sub-network derived from MIPS (MAPK MIPS) shows 37 genes and 74 interactions and the sub-network from ALL HC (MAPK allhc) shows 39 genes and 106 interactions.

Figure 5. The result of the query on the high-confidence MAPK subgraph. Each edge between two nodes comes from a different source. Nodes are colored according to GO annotations for bio- logical process at level 5; purple: protein metabolism and modification, dark green: polar budding, light green: cell cycle regulation, yellow: signal transduction, light yellow: cell surface receptor linked signal transduction, aqua: perception of external stimulus, teal: cellular organization and biogenesis, dark pink: DNA replication and chromosome cycle, light pink: cytoplasm organization and biogenesis, magenta: growth pattern, orange: M phase, light orange: cell surface organization and biogenesis, dark blue: nucleic acid metabolism, light blue: sporulation, grey: vesicle mediated trans- port, lavender: Not annotated. Edges are colored according to interaction type; red: MIPS physical, blue: yeast two-hybrid, purple: co- immunoprecipitation, green: MIPS genetic interaction, pink: DNA- protein interaction (MIT).
